# Comparative Investigation of the Differences in Chemical Compounds between Raw and Processed *Mume Fructus* Using Plant Metabolomics Combined with Chemometrics Methods

**DOI:** 10.3390/molecules27196344

**Published:** 2022-09-26

**Authors:** Songrui Wang, Shujie Wei, Yameng Zhu, Mengmeng Zhang, Xiunan Cao, Yanxu Chang, Huizi Ouyang, Jun He

**Affiliations:** State Key Laboratory of Component-Based Chinese Medicine, Tianjin University of Traditional Chinese Medicine, Tianjin 301617, China

**Keywords:** *Mume Fructus*, processing, plant metabolomics, chemometrics, quality evaluation

## Abstract

*Mume Fructus* is a well-known herbal medicine and food with a long history of processing and application. Different processing methods impact the intrinsic quality of *Mume Fructus*. Thus, it is of great significance to investigate the changes in chemical components during processing (i.e., raw compared to the pulp and charcoal forms). In this study, plant metabolomics methods based on mass spectrometry detection were established to analyze the chemical ingredients of *Mume Fructus* comprehensively. Chemometric strategies were combined to analyze the profile differences of *Mume Fructus* after different processing methods. The established strategy identified 98 volatile and 89 non-volatile compounds of *Mume Fructus* by gas chromatography-mass spectrometry (GC-MS) and ultra-high performance liquid chromatography coupled with quadrupole time of flight mass spectrometry (UHPLC-Q-TOF-MS/MS), respectively. Moreover, the orthogonal partial least squares discriminant analysis (OPLS-DA) indicated that raw *Mume Fructus* and the *Mume Fructus* pulp and charcoal were distributed in three regions. Subsequently, 19 volatile and 16 non-volatile components were selected as potential chemical component markers with variable importance in the projection using (VIP) >1 as the criterion, and the accuracy was verified by a Back Propagation Neural Network (BP-NN). To further understand the difference in the content of *Mume Fructus* before and after processing, 16 non-volatile chemical component markers were quantitatively determined by ultra-high performance liquid chromatography-mass spectrometry (UHPLC-MS/MS). The results revealed that, compared with raw *Mume Fructus*, the total content of 16 components in the pulp of *Mume Fructus* increased while it decreased in the charcoal. Therefore, this study used GC-MS, UHPLC-Q-TOF-MS/MS and UHPLC-MS/MS modern technology to analyze the differences in chemical components before and after the processing of *Mume Fructus* and provided a material basis for further research on the quality evaluation and efficacy of *Mume Fructus*.

## 1. Introduction

*Mume Fructus* (MF) is derived from the immature fruit of *Prunus mume* Sieb. etZucc [1]. It is also known as wumei in China, Japanese apricot or ume in Japan, and maesil or oumae in Korea. The plant is native to Japan and South Korea and is widely planted in Yunnan, Sichuan, Xinjiang, and other regions in China [2]. As a common commercial food, it is also used to prepare plum sauce, plum juice, and plum wine, which can be consumed as snacks, condiments, or food additives. Phytochemical studies have shown that MF contains various chemical components, including non-volatile and volatile components. The research on non-volatile components mainly focuses on organic acids, flavonoids, terpenoids, amino acids, polysaccharides, and nucleotides. The organic acids are one of the main active components, and the citric acid content was used as a detection index for quality control of MF in the 2020 Chinese Pharmacopoeia [3,4,5,6,7]. Modern pharmacological studies indicated that these chemical ingredients could have a variety of biological activities, such as antibacterial, antitumor, antiulcer, antivirus, antioxidant, and antifertility activities [5,8,9].

Processing is a unique pharmaceutical skill of traditional Chinese medicine (TCM), which can promote therapeutic effects or reduce toxic ingredients and their side effects [10]. Generally speaking, most Chinese medicines should be prepared by special processing methods such as steaming, boiling, frying, and stewing before clinical use [11]. A series of chemical reactions such as oxidation, hydrolysis, and isomerization would occur during the processing, which leads to changes in the content and type of some chemical components [12,13,14,15]. In recent years, most of the MF foods sold in the market were processed, and both MF and its processed products were widely used to treat diseases in clinics. Raw MF has obvious antitussive effect but the MF pulp does not, and its antitussive effect may be related to amygdalin contained in MF [16,17]. The MF pulp and charcoal could reduce the level of blood glucose in normal mice, and their hypoglycemic effect is related to malic acid and citric acid in MF [18]. Moreover, raw MF has no coagulation effect, and the content of tannins and organic acids decreased after frying with charcoal, and the MF charcoal can increase the effect of hemostasis [19,20]. However, few studies have reported the difference in ingredients between raw and processed MF samples.

Metabolomics has been widely used to study the changes of secondary metabolites, understand metabolic networks, discover biomarkers, and assess the quality of TCMs [21]. Plant metabolomics is an important branch of metabolomics based on index analysis of groupings. It can be used to analyze the differences in chemical composition in different environments or facilitate the discovery of differential markers [22]. A variety of metabolite detection methods have been developed, such as ultra-high performance liquid chromatography coupled with time-of-flight mass spectrometry (UHPLC-Q-TOF-MS/MS), liquid chromatography-mass spectrometry (LC-MS), gas chromatography-mass spectrometry (GC-MS), and nuclear magnetic resonance (NMR), and UHPLC-Q-TOF-MS/MS has become a powerful tool for metabolomics research. It has the advantages of a short analysis time, good specificity, and high selectivity and sensitivity [23,24,25,26,27]. Chemometrics is a method of combining mathematics and statistics. It can provide various algorithms to help obtain useful chromatographic data and extract qualitative, quantitative, and structural information by analyzing the data of related substances [28,29]. Chemometrics analysis technology could objectively analyze the data obtained from various modern instruments such as HPLC, UHPLC-Q-TOF-MS/MS, IR, and NMR. It can carry out statistical analysis on multiple indicators and quantify information from the entire spectrum so that it can be recognized and processed by a computer. It can reflect the information more objectively and achieve comprehensive quality control of traditional Chinese medicine [30,31].

In this study, a plant metabolomics method based on mass spectrometry detection was established to analyze the chemical components of MF comprehensively. We combined this with a chemometrics strategy to assess the differences before and after the processing of MF. The volatile and non-volatile components in MF were identified by GC-MS and UHPLC-Q-TOF-MS/MS methods, respectively. Furthermore, OPLS-DA was used to screen out volatile and non-volatile components as potential chemical component markers with VIP > 1 as the criterion, and BP-NN verified the accuracy. To further understand the difference in the content of MF before and after processing, the potential non-volatile chemical markers were quantitatively determined using UHPLC-MS/MS, and the validity of the biomarkers was verified by discriminant analysis. The overall chemical composition differences of MF before and after processing were discussed, and the basis for the quality evaluation and clinical application of MF was provided.

## 2. Results and Discussion

### 2.1. Volatile Ingredients Analysis

#### 2.1.1. Method Validation

The GC-MS method was verified in terms of precision, stability, and repeatability. The RSDs of the retention time and peak area were less than 0.65% and 9.12%, as shown in Supplementary Table S1, suggesting that the GC-MS method was precise for analyzing MF samples.

#### 2.1.2. Volatile Ingredient Identification

**Figure 1:**
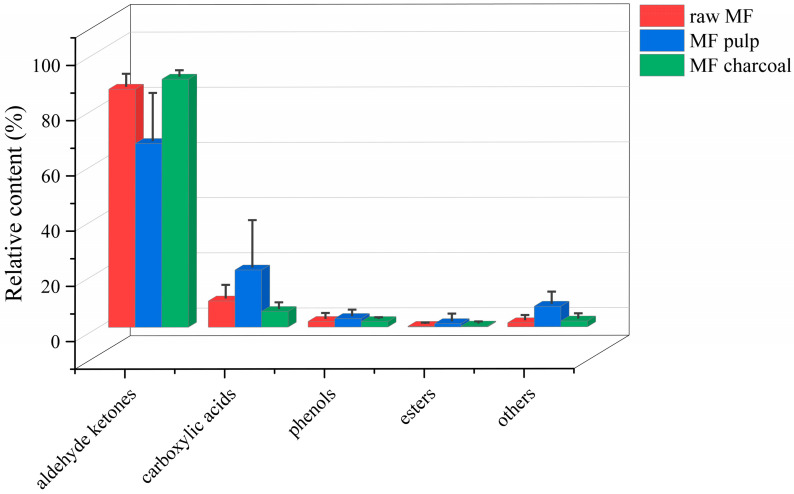
The relative contents of all kinds of compounds in different processed *Mume Fructus* (MF) samples with GC-MS analysis.

The total ion chromatogram (TIC) of the MF samples is provided in Supplementary Figure S1. Based on the automatic peak identification procedures, the volatile compounds were identified against the GC-MS NIST08 and NIST08s databases. Compounds were identified with a match similarity higher than 75%, and the peak area data were obtained by peak area integration and expressed as a relative content using the area normalization method. A total of 98 compounds (Table 1) were detected in different processed MF samples, mainly aldehyde ketones, phenols, carboxylic acids, and esters. The compounds in the MF pulp were the most diverse, up to 68, whereas raw MF and MF charcoal had 53 and 44 components, respectively. After the MF is processed, the relative content of volatile compounds of raw MF and MF pulp and charcoal differed, as shown in Figure 1. The aldehyde ketones and carboxylic acids have a high content in all samples, while the esters contents were low. The carboxylic acids, phenols and esters of raw MF exhibited an increasing trend by removing the core. The aldehydes ketones were increased after the raw MF was processed into charcoal. In general, after removing the core, the types of volatile components of MF increased, while the types of volatile components of MF charcoal decreased, which was related to the chemical and physical changes during the charcoal frying process, such as reduction and oxidation reactions.

#### 2.1.3. Plant Metabolomics Analysis and Identification of Volatile Chemical Markers

**Figure 2:**
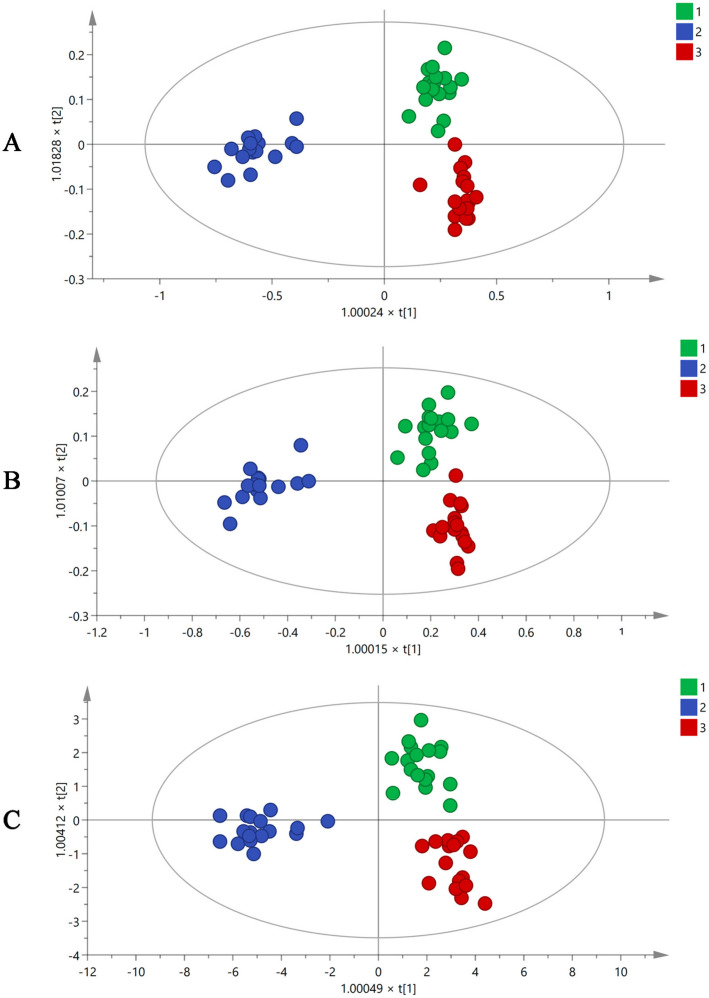
The OPLS-DA figures of three groups MF samples using GC-MS analysis by 487 variables (**A**), 99 variables (**B**), and 19 variables (**C**) (1. raw MF; 2. MF pulp; 3. MF charcoal).

Plant metabolomics analysis has been used to determine different chemical compositions in different environments. Using the XCMS online data analysis platform, all mass spectrum data obtained by GC-MS were converted to a three-dimensional matrix containing Rt, *m*/*z*, and peak intensity information. A total of 487 variables were acquired and imported to SIMCA-P 14.1 for multiple statistical analyses. Orthogonal partial least squares discriminant analysis (OPLS-DA), as a supervised multivariate analysis method, can eliminate differences between groups, exclude irrelevant variations, and make it easier to identify system information and noise. The OPLS-DA results (Figure 2A) showed that raw MF and MF pulp and charcoal were clearly distributed in different regions using 487 variables, and the volatile ingredients of the three groups had clear differences. However, using 487 variables to differentiate three groups of MF samples was difficult. Therefore, the different contributions were analyzed to obtain the variable importance in projection value (VIP) based on OPLS-DA analysis. The components with VIP > 1 were used for the subsequent analysis. Then, 99 of the 487 variables were screened, as shown in Figure 2B, and the raw and processed MF samples were well distinguished through these 99 variables.

The identification of the compound was mainly based on accurate molecular mass, retention time, and MS/MS information. The potential markers were determined against the NIST08 and NIST08s databases. Finally, 19 differential volatile chemical markers were identified from 99 variables. They were compounds 1, 4, 7, 8, 10, 11, 13, 17, 19, 26, 33, 35, 36, 39, 41, 73, 85, 88 and 90 (Table 1). The OPLS-DA diagrams (Figure 2C) demonstrated that three groups of MF samples could be distinguished using 19 potential markers. The compounds with loadings that were distant from the origin on the OPLS-DA loading plots (Supplementary Figure S2) were inferred to make the greatest contribution to class separation, 19 differential volatile chemical markers were major contributors to the separation among the raw and processed MF samples. In the meantime, the accuracy of the selected differential compounds should be further verified. BP-NN, a supervised learning model, was used to determine the accuracy of each step’s variables. The batches of R1–11, P1–11, and C1–11 were set as the raw MF and MF pulp and charcoal training sets. The batches R12–14 of raw MF, P12–14 of MF pulp, and C12–14 of MF charcoal were identified as the validation sets. The remaining batches (R15–17, P15–17, and C15–17) were defined as the testing sets. The results (Supplementary Table S2) showed that the accuracy of all variables exceeded 85%, indicating that the 19 differential components could represent volatile compounds in MF to distinguish the raw MF and MF pulp and charcoal samples.

### 2.2. Non-Volatile Components Analysis

#### 2.2.1. UHPLC-Q-TOF-MS/MS Acquisition Method Validation

The retention time and peak area of the twenty selected chromatographic peaks were used to calculate the RSD values, which were considered an important evaluation index for precision, repeatability, and stability. The RSD of the precision values was all below 6.75%, indicating that the method has high accuracy. The repeatability of the RSDs ranged from 0.05–6.88%, demonstrating the consistency of the results of the method. The RSDs of stability were within 0.01–6.99%, which illustrated that the sample solution was stable over 24 h. All the above results (Supplementary Table S3) displayed that the UHPLC-Q-TOF-MS/MS method was reliable for the plant metabolomics data.

#### 2.2.2. Compound Identification in *Mume Fructus*

The identification of compounds was crucial for screening candidate markers in the subsequent studies. The plant metabolomics data of raw and processed MF samples was acquired in both positive and negative ESI modes, and the TIC figures are illustrated in Supplementary Figure S3. The obtained mass spectrograms were verified by: (a) matching with the molecular formula generated by the instrument; (b) analyzing the compound information acquired from the Metlin database (http://metlin.scripps.edu, accessed on 29 June 2022); (c) comparing with the fragment information of the standard products; (d) taking reference to the compound information of previous reports. The requisite criteria were applied, which are exact mass-to-nucleus ratio of the precursor ions within an error of 10 ppm, and then inferred the chemical composition based on the fragment ions and the structural formula of the compound. By the above-mentioned data acquisition and mining strategies, 89 compounds, mainly organic acids, amino acids, flavonoids, and triterpenes, were tentatively identified. The detailed information on the composition is shown in Table 2.

As listed in Table 2, compounds were identified based on their characteristic MS fragmentation patterns compared to references and standards. Taking the fragmentation process of compound 13 (organic acid) as an example, it exhibited an [M−H]^−^ ion at *m*/*z* 191.0157 (C_6_H_8_O_7_) and yielded fragment ions at *m*/*z* 173.0045, 129.0159 and 111.0051 by the successive losses of H_2_O and CO_2_. It can be inferred to be Citric acid by comparison with literature and standard material. Compound 17 (amino acid) gave an [M+H]^+^ ion at *m*/*z* 166.0895 (C_9_H_11_NO_2_) in the positive ion mode, then it lost a molecule of NH_3_ and COOH to form a [M+H−COOH]^+^ fragment ion of *m*/*z* 120.0807 and [M+H−NH_3_−COOH]^+^ fragment ion of *m*/*z* 103.0544, compared with the relevant reference, it was determined to be phenylalanine.

#### 2.2.3. Plant Metabolomics Data Analysis and Verification of Differential Markers

**Figure 3:**
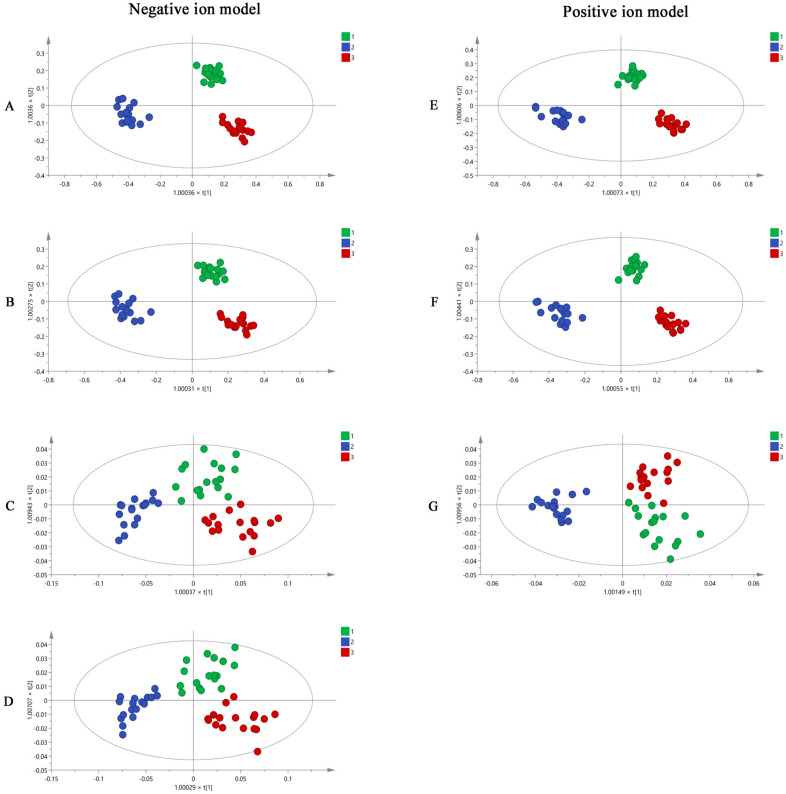
The OPLS-DA figures of three groups MF samples using UHPLC-Q-TOF-MS/MS analysis by 2986 variables (**A**), 420 variables (**B**), 26 variables (**C**), 16 variables (**D**) in negative ion model; and 3605 variables (**E**), 674 variables (**F**), 12 variables (**G**) in positive ion model (1. raw MF; 2. MF pulp; 3. MF charcoal).

R software and SIMCA software were used to analyze the UHPLC-Q-TOF-MS/MS results. Using the R software, all the mass spectrometry data of MF samples obtained from UHPLC-Q-TOF-MS/MS were converted into a three-dimensional matrix, including retention time (Rt), *m*/*z* value, and peak intensity. Then, 2986 and 3605 variables were obtained in negative and positive ion modes and were used in OPLS-DA analysis in the SIMCA software. The OPLS-DA diagrams (Figure 3A,E) showed that raw MF and MF pulp and charcoal samples were distributed in three different regions using 2986 and 3605 variables, suggesting that the processed methods impact the chemical composition of MF. However, using this volume of variables to distinguish the MF samples is impractical. Thus, the substances with VIP > 1 were used as potential difference markers for subsequent analysis of the MF samples. A total of 420 and 674 variables were filtered from the 2986 and 3605 variables, respectively. The raw MF and MF pulp and charcoal samples were distinguished well using the 420 and 674 variables (Figure 3B,F).

The 420 and 674 variables with VIP > 1 were accurately identified based on Rt, *m*/*z*, and fragment information. From this, 26 and 12 compounds were accurately identified. They were compounds 1, 3, 5, 6, 7, 8, 9, 10, 11, 13, 14, 15, 16, 19, 22, 26, 28, 29, 30, 31, 33, 35, 36, 37, 38, 39, 40, 41, 48, 60, 61, 63, 76, 81, 85, 86, 87, and 89 (Table 2). In addition, the OPLS-DA results (Figure 3C,G) indicated that the 38 components have the potential to distinguish raw MF and MF pulp and charcoal samples. To easily quantify and quickly distinguish the three types of MF samples, 16 compounds (succinic acid, L-malic acid, 3,4-Dihydroxybenzaldehyde, protocatechuic acid, caffeic acid, D-quinic acid, citric acid, ferulic acid, syringic acid, cryptochlorogenic acid, neochlorogenic acid, chlorogenic acid, amygdalin, maslinic acid, corosolic acid, and rutin) were selected as potential differential markers. The OPLS-DA figures (Figure 3D) suggested that 16 markers could separate the raw MF and MF pulp and charcoal samples. The OPLS-DA loading plot showed the variables that contributed to the separation on MF samples (Supplementary Figure S4). However, the accuracy of the selected variables was unknown. Therefore, BP-NN was used to predict the accuracy of each step to generate variables. The training, validation, and testing sets were defined as the same GC-MS analysis. The results (Supplementary Table S2) showed that the accuracies of all variables in the positive and negative ion modes were higher than 79%. Interestingly, the accuracy of 16 variables was equivalent to using 420 variables and even close to using 2986 variables. In conclusion, the 16 compounds could be used for the quality evaluation of MF samples.

#### 2.2.4. UHPLC-MS/MS Quantitative Method Validation

Quantitative method validation of the established UHPLC-MS/MS method was performed to determine linearity, LLODs (Lower Limit of Detections), LLOQs (Lower Limit of Quantitations), intra- and inter-day precision, repeatability, stability, recovery, and the dilution effect. The results were displayed in Supplementary Tables S4–S6. The correlation coefficient values (r ≥ 0.9991) for the 16 constituents indicated good linearity within the concentration range. The range of LLOQs and LLODs were from 0.13–40.19 ng/mL and 0.04–12.06 ng/mL, respectively. The RSDs of intra- and inter-day precisions of 16 analytes were within 0.63–6.77% and 1.06–6.07%, respectively. The method could determine multiple samples due to the RSDs of repeatability of less than 5.90%. As for stability, the RSDs were lower than 6.90%. The results indicated that the quantitative method could accurately determine the samples over several days. The developed method also had acceptable accuracy, recovering 88.81–110.45% of all compounds. The RSD values of the dilution effect were less than 6.94%, and the RE ranged from −7.31–5.69%, indicating that the content measured was accurate when the samples were diluted within a certain range. In general, the established UHPLC-MS/MS method was suitable for analyzing 16 components in the MF samples. The analyte’s multiple reaction monitoring (MRM) diagram is shown in Supplementary Figure S5.

#### 2.2.5. Analysis of Different Processed Methods of *Mume Fructus* Samples

**Figure 4:**
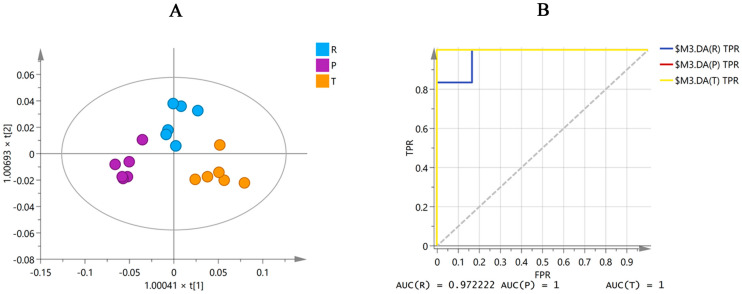
The OPLS-DA figure (**A**) and ROC curve (**B**) of three groups MF samples in Sichuan province by 16 differential markers (R, raw MF; P, MF pulp; C, MF charcoal). AUC in the figure represents the area under the ROC curve.

Six batches of raw MF and MF pulp and charcoal samples from Sichuan province were analyzed using the same OPLS-DA analysis with the 16 differential markers to eliminate the impact of origin on the quality markers. The results (Figure 4A) showed that the 16 compounds could divide the MF samples into three groups, including raw MF and MF pulp and charcoal. Moreover, the ROC curve was generated to verify the classification capabilities of the model. As shown in Figure 4B, the ROC curve passed through the left upper corner and AUC (the region under the ROC curve) close to 1, suggesting that the 16 markers could accurately classify these three groups of MF samples. Therefore, processing could alter the content of the 16 compounds in the raw MF samples, leading to differences between the raw MF samples and the MF pulp and charcoal samples.

The validated UHPLC-MS/MS method was used to simultaneously determine the 16 active compounds (succinic acid, L-malic acid, 3,4-Dihydroxybenzaldehyde, protocatechuic acid, caffeic acid, D-quinic acid, citric acid, ferulic acid, syringic acid, cryptochlorogenic acid, neochlorogenic acid, chlorogenic acid, amygdalin, maslinic acid, corosolic acid, and rutin) in raw MF and MF pulp and charcoal samples. The contents of 16 components in MF samples are presented in Supplementary Table S7.

**Figure 5:**
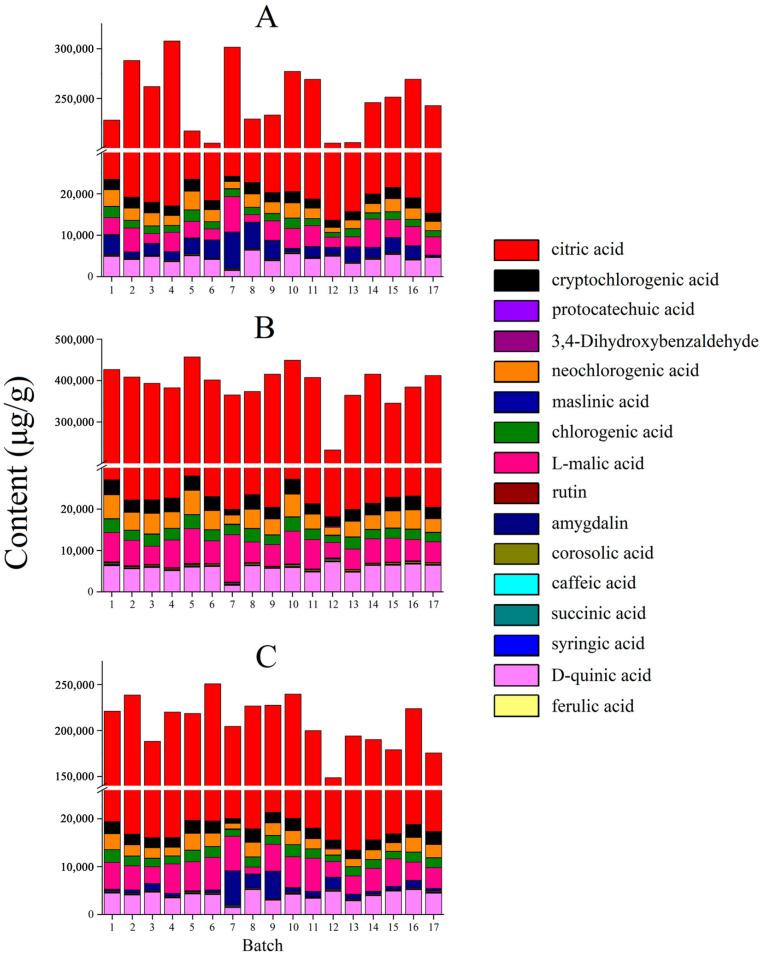
The contents of 16 compounds in MF samples of different processed methods (μg/g): (**A**) raw MF; (**B**) MF pulp; (**C**) MF charcoal.

As shown in Figure 5, there were differences in the total content of 16 components in the two processing methods compared to that of raw MF. Compared with raw MF, the total content of organic acids in the MF pulp was higher, showing that the organic acids are mainly located in the pulp. The pharmacological effects of raw MF and MF pulp are similar, but the efficacy of MF pulp is stronger, which may be related to the higher content of organic acids in MF pulp. And the organic acid content in MF charcoal is the lowest, indicating that heating and drying during charcoal production can reduce the acidity of raw MF, which is the same as the statement that “the damage to the teeth can be avoided after MF charcoal” [32]. In terms of individual components, the citric acid content of the MF charcoal was significantly lower than in the raw MF (*p* < 0.01), indicating that it can be broken down into other products under high temperature conditions. Compared with raw MF, the amygdalin content of the MF pulp was lower, which may be attributed to the presence of amygdalin mainly in the core shell and kernel. The raw MF have obvious antitussive effect, but the MF pulp has no antitussive effect, which may be related to the low content of amygdalin in the pulp [16,17]. Moreover, the content of amygdalin in MF charcoal was significantly reduced (*p* < 0.01), revealing that heating conditions may accelerate the isomerization and decomposition of amygdalin.

#### 2.2.6. Discriminant Analysis

Discriminant analysis was used to predict the classification of raw MF and MF pulp and charcoal in unknown samples. The raw MF (R1–R12), MF pulp (P1–P12), and MF charcoal (C1–C12) were marked as group 1, group 2, and group 3 (Table 3), respectively. The contents of 16 components of these samples were used as modeling data to construct a discriminant analysis model using SPSS software. The discriminant function equations of the MF samples were as follows (S1: succinic acid, S2: L-malic acid, S3: 3,4-Dihydroxybenzaldehyde, S4: protocatechuic acid, S5: caffeic acid, S6: D-quinic acid, S7: citric acid, S8: ferulic acid, S9: syringic acid, S10: cryptochlorogenic acid, S11: neochlorogenic acid, S12: chlorogenic acid, S13: amygdalin, S14: maslinic acid, S15: corosolic acid, S16: rutin):

Y1 = 0.781S1 + 0.016S2 − 0.447S3 + 0.746S4 − 1.469S5 + 0.046S6 + 0.001S7 + 1.239S8 − 0.107S9 + 0.122S10 − 0.042S11 + 0.147S12 + 0.015S13 + 0.192S14 − 0.627S15 − 1.328S16 − 358.309 (Raw MF)

Y2 = 0.891S1 + 0.017S2 − 0.659S3 + 0.849S4 − 2.255S5 + 0.055S6 + 0.001S7 + 0.607S8 − 0.383S9 + 0.108S10 − 0.027S11 + 0.155S12 + 0.013S13 + 0.315S14 − 0.481S15 − 1.330S16 − 563.057 (MF Pulp)

Y3 = 0.803S1 + 0.019S2 − 1.377S3 + 0.980S4 − 1.423S5 + 0.043S6 + 0.001S7 + 1.769S8 − 0.726S9 + 0.147S10 − 0.053S11 + 0.176S12 + 0.017S13 + 0.206S14 − 0.804S15 − 1.531S16 − 425.465 (MF Charcoal)

The content of each chromatographic peak of different batches of MF samples was used in the functional equation to obtain the Y value. We tested 15 batches of MF samples of known origin (R13–R17, P13–P17, and C13–C17) using the obtained discriminant function, and the discriminant analysis results were compared with the actual sources, as shown in Table 3. The results indicated that most MF samples were correctly classified, only one sample (C15) was incorrectly predicted, and the classification model’s accuracy model was 93%. This demonstrated that simultaneous determination of 16 components combined with discriminant analysis could accurately predict the classification of raw MF and MF pulp and charcoal samples.

## 3. Materials and Methods

### 3.1. Sample Collection

A total of 51 batches of raw and processed MF samples were used in this study. Among them, 17 batches of raw MF (Supplementary Table S8) were collected from May to July 2020 in four provinces (Yunnan, Sichuan, Xinjiang, and Anhui) of China. Moreover, according to Chinese Pharmacopeia (2020 edition), we processed 17 batches of MF pulp (P1–P17) and charcoal (C1–C17) using the raw MF (R1–R17).

### 3.2. Processing Methods of Mume Fructus

MF Pulp: the raw MF samples were pressed, the pulp was taken out, and dried in a heating-air drying oven at approximately 50 °C. MF Charcoal: take the raw MF samples and put them in a metallic pan, heat them with a strong fire, fry until black outside and brown inside, take them out and dried in a heating-air drying oven at approximately 50 °C.

### 3.3. GC-MS Analysis

#### 3.3.1. Apparatus

The volatile components were analyzed by a QP 2010 GC-MS (Shimadzu, Kyoto, Japan), equipped with an HSS 86.50 headspace sampler and AOC-20i autosampler.

#### 3.3.2. Sample Preparation and Measurement

All batches of raw MF and MF pulp and charcoal were dried and pulverized to finer than 60 mesh; a 2.0 g sample was then sealed in the headspace bottle (20 mL) for analysis. The heating box, quantitative ring, and transmission line temperatures were 100 °C, 120 °C, and 140 °C, respectively. The equilibrium time was 20 min, and the injection time was 1 min.

Chromatographic separation was achieved on a DB-17 column (0.25 mm × 30 m × 0.25 µm). The initial oven temperature was set at 80 °C, warmed to 200 °C at 10 °C/min, 210 °C at 2 °C/min, 260 °C at 6 °C/min, and then maintained for 10 min. The injector temperature was 250 °C. High purity helium was used as carrier gas at a flow rate of 1 mL/min. The injection volume was 1 mL with a 20:1 split ratio. MS detection was performed with an electronic bombardment source in full scan mode at *m*/*z* 20–700. The ion source and interface temperatures were 230 °C and 250 °C, respectively. The detector voltage was 1.3 kV.

#### 3.3.3. Method Validation

The precision, repeatability, and stability of GC-MS analysis were verified using the raw MF (batch 8) sample. Six consecutive injections of one sample were measured on the same day for intra-day variance assessment. The repeatability was determined by preparing six replicate samples, and one of the samples was tested at 0, 2, 4, 8, and 12 h for stability. Twenty chromatographic peaks (compounds 7, 8, 11, 17, 18, 20, 26, 28, 33, 39, 46, 48, 49, 64, 68, 73, 75, 80, 85 and 86 in Table 1) were selected to calculate relative standard deviation (RSD) values.

#### 3.3.4. Data Pre-Processing

The collected data were converted into MZ data by GC-MS Postrun Analysis (Shimadzu, Kyoto, Japan). The data of all batches of raw and processed MF samples were introduced to R 2.7.2 software (R Foundation for Statistical Computing, Vienna, Austria) to obtain a three-dimensional matrix including retention time (Rt), mass/charge ratio (*m*/*z*), and peak intensities. The data obtained was imported into SIMCA-P 14.1 statistical software (Umetrics AB, Umea, Sweden) for multivariate statistical analysis to screen differential markers. The selected differential components’ accuracy was calculated by the BP-NN algorithm using Matlab R2014a (Mathworks, Natick, MA, USA).

### 3.4. UHPLC-Q-TOF-MS/MS Analysis

#### 3.4.1. Chemicals and Apparatus

Chromatographic grade acetonitrile and methanol were purchased from Thermo Fisher Scientific Co., Ltd. (Waltham, MA, USA). HPLC-grade formic acid was provided by ROE (St. Louis, MO, USA). Deionized water was purified using a Milli-Q purification system (Millipore, Milford, MA, USA). The standards (i.e., citric acid, L-malic acid, succinic acid, D-quinic acid, syringic acid, caffeic acid, chlorogenic acid, cryptochlorogenic acid, neochlorogenic acid, corosolic acid, protocatechuic acid, maslinic acid, rutin, ferulic acid, and 3,4-Dihydroxybenzaldehyde) were prepared from Chengdu Desite Co., Ltd. (Chengdu, China). Amygdalin was obtained from the National Institutes for Food and Drug Control (Beijing, China).

The UHPLC-Q-TOF-MS/MS system consisted of an Agilent 1290 UHPLC instrument (Agilent Technologies Inc., Palo Alto, CA, USA) and an Agilent 6520 Q-TOF mass spectrometer (Agilent Corporation, Santa Clara, CA, USA).

#### 3.4.2. Sample Preparation and Measurement

The standards (syringic acid, caffeic acid, chlorogenic acid, cryptochlorogenic acid, neochlorogenic acid, corosolic acid, maslinic acid, rutin, ferulic acid, 3,4-Dihydroxybenzaldehyde, amygdalin) were accurately weighed and dissolved with methanol solvent at a concentration of 1 mg/mL. Citric acid, L-malic acid, succinic acid, D-quinic acid, and protocatechuic acid were prepared in water at a concentration of 1 mg/mL. The individual standard solutions were mixed as a stock solution and further diluted with methanol to a working standard.

All batches of MF samples were dried, powdered and passed through a 60 mesh, 0.3 mm aperture sieve. Pulverized samples (1 g) were accurately weighed and then extracted in an ultrasonic bath (40 kHz, 180W) for 40 min at 25 ± 2 °C with 25 mL 80% methanol in water. All sample solutions were centrifuged at 14,000 rpm for 10 min, and the supernatant was filtered through a 0.22 μm membrane.

Chromatographic separation was achieved on an ACQUITY UPLC^®^HSS T3 column (2.1 × 100 mm, 1.8 µm, Waters) held at 30 °C, and the flow rate was 0.2 mL/min. The mobile phases consisted of 0.1% formic acid-water (A) and acetonitrile (B) with a gradient elution as follows: 0–10 min, 5–10% B; 10–20 min, 10–15% B; 20–30 min, 15–30% B; 30–45 min, 30–95% B; 45–53 min, 95% B. The injection volume was 2 µL. The mass spectra data was acquired in both positive and negative ion modes. The optimal Q-TOF/MS parameters were as follows: drying gas flow, 11 L/min; capillary temperature, 350 °C; nebulizer pressure, 40 psi; fragmentor voltage, 135 V; and collision energy, 40 V. The scan range of mass spectra was *m*/*z* 50–2000.

#### 3.4.3. Method Validation

The precision, repeatability, and stability were used to verify the applicability of the UHPLC-Q-TOF-MS/MS method by using the raw MF (batch 8) sample. Twenty chromatographic peaks (compounds 3, 5, 13, 14, 22, 31, 33, 36, 37, 39, 40, 41, 48, 59, 61, 66, 69, 73, 86 and 87 in Table 2) were selected to calculate RSDs in order to verify precision, repeatability, and stability.

#### 3.4.4. Data Pre-Processing

The UHPLC-Q-TOF-MS/MS plant metabolomics data were converted into MZ data using Agilent Masshunter Qualitative Workstation Analysis B.07.00 (Agilent Technologies Inc., Santa Clara, CA, USA). The data were then imported to R software and SIMCA-P 14.1 software for further analysis as the processing of GC-MS analysis.

### 3.5. UHPLC-MS/MS Analysis

#### 3.5.1. Chemicals and Apparatus

The quantitative analysis was carried out on an Agilent 1290 UHPLC instrument (Agilent Technologies, Waldbronn, Germany) coupled with an Agilent 6470 series triple quadrupole mass spectrometer (Agilent Technologies, Singapore, Singapore). The same chemicals prepared for the UHPLC-Q-TOF-MS/MS analysis were used.

#### 3.5.2. Sample Preparation and Measurement

The standard solution preparation was the same as described in Section 3.4.2. The quantitative sample powder was accurately weighed (50 mg), and the subsequent ultrasound step was the same as for the UHPLC-Q-TOF-MS/MS analysis. The sample solution was diluted 50 times to determine citric acid.

The chromatographic peaks were separated on an ACQUITY UPLC^®^BEH C18 column (2.1 × 100 mm, 1.7 µm, Waters) at 20 °C with a flow rate of 0.3 mL/min. Mobile phases consisted of 0.1% formic acid-water (A) and methanol (B). The gradient elution was: 0–5 min, 10–40% B; 5–5.5 min, 40–80% B; 5.5–7 min, 80–83% B; and 7–14 min, 83% B. The injection volume was 2 µL. Electron Spray Ionization (ESI) source and multiple reaction monitoring (MRM) mode were used to obtain mass spectrometry data in the negative ion mode. The optimum MS settings were maintained as follows: gas temperature, 300 °C; gas flow, 7 L/min; nebulizer, 35 psi; sheath gas temperature, 350 °C; sheath gas flow, 11 L/min; capillary voltage, 3500 V. The optimized MRM parameters are shown in Table 4.

#### 3.5.3. Method Validation

Stock solutions containing 16 standard compounds were prepared and diluted to a series of appropriate concentrations to construct the calibration curve. The linearity for each compound was determined by weighted (1/X) least-squares linear regression of the standard peak areas (Y) against the normalized standard concentrations (X). Under the present chromatographic conditions, lower limits of detections (LLODs) and quantifications (LLOQs) were detected by diluting the standard solution when the signal-to-noise ratios (S/N) were approximately 3 and 10, respectively. The raw MF sample (batch 8) was used to validate the method, including precision, repeatability, stability, and recovery. The dilution effect was verified using a known concentration of the standard solution. The intra- and inter-day precisions were determined by analyzing six replicates on three consecutive days. Six independent samples of the raw MF (batch 8) were extracted and analyzed to determine the repeatability. The stability test was obtained using one sample solution stored at 25 ± 2 °C and analyzed at 0, 2, 4, 6, 12, and 24 h. The recovery test was used to evaluate the accuracy of this method. A certain amount of 16 standards mixture was added to six accurately weighed (25 mg) samples of raw MF (batch 8) and extracted using the methods mentioned above. The recovery was calculated according to the following equation:

recovery (%) = (determined amount − original amount)/spiked amount × 100%.

The dilution effect was evaluated by mixing standard solutions of known concentrations, using 1:20, 1:50, and 1:100 dilution factors. The accuracy was assessed according to the equation:

Relative Error (RE, %) = (measured concentration − theoretical concentration)/theoretical concentration × 100%.

All of the above variations were assessed by RSDs.

#### 3.5.4. Data Analysis

The UHPLC-MS/MS method was employed to determine the content of partially differential markers simultaneously. The compound content data was imported into SPSS 21.0 (IBM, San Diego, CA, USA) for discriminant analysis to predict the classification of unknown samples.

## 4. Conclusions

In this study, a GC-MS and UHPLC-Q-TOF-MS/MS plant metabolomics method were applied to reflect the general characteristics of MF. A chemometrics strategy was used to distinguish the MF samples from different processing methods. According to the OPLS-DA diagrams of volatile and non-volatile components, the raw MF and MF pulp and charcoal samples were classified into three groups, indicating that the processing method greatly influenced the MF samples. A total of 98 volatile compounds were identified, and 19 constituents with a VIP > 1 were selected as potential markers in GC-MS analysis. Through UHPLC-Q-TOF-MS/MS analysis, 89 compounds were identified, and 16 were selected as quality control markers to distinguish the MF samples. Furthermore, UHPLC-MS/MS analysis was used for quantitative analysis of the 16 differential chemical components, and the discriminant analysis showed that the quantification of the above components can accurately distinguish MF samples with different processing methods. In conclusion, the developed plant metabolomics method coupled with a chemometrics strategy was helpful for screening quality markers for distinguishing the raw MF and MF pulp and charcoal samples, and it would provide a reliable reference for the development of TCM or other related food and drug.

## Figures and Tables

**Table 1:** Volatile chemical components identification of *Mume Fructus* (MF) samples.

No.	Compound	Molecular Formula	Similarity			Relative Content (%)		
			Raw MF	MF Pulp	MF Charcoal	Raw MF	MF Pulp	MF Charcoal
1	Acetol	C_3_H_6_O_2_	82	-	-	0.02	-	-
2	(E)-2-Pentenal	C_5_H_8_O	87	-	-	0.04	-	-
3	3-Pyrroline	C_4_H_7_N	-	84	-	-	0.11	-
4	Pentanol	C_5_H_12_O	93	94	93	0.20	0.21	0.13
5	3-methylpent-4-en-1-ol	C_6_H_12_O	81	-	-	0.05	-	-
6	2-Ethyl-1-butanol	C_6_H_14_O	80	87	-	0.10	0.10	-
7	Hexanal	C_6_H_12_O	91	95	90	0.73	1.92	0.45
8	3-Methylbutanoic acid	C_5_H_10_O_2_	89	81	84	0.12	0.15	0.39
9	2-Methylbutyric acid	C_5_H_10_O_2_	-	80	91	-	0.16	0.11
10	1,3-octadiene	C_8_H_14_	-	94	-	-	0.08	-
11	Furfural	C_5_H_4_O_2_	96	96	97	7.02	5.77	8.02
12	1-ethyl-2-methylcyclopentene	C_8_H_14_	85	-	-	0.15	-	-
13	Acethydrazide	C_2_H_6_N_2_O	80	-	-	0.10	-	-
14	Cyclohexenone	C_6_H_8_O	85	-	-	0.12	-	-
15	Propylene carbonate	C_4_H_6_O_3_	83	-	-	0.02	-	-
16	Tetrahydro-4-pyranol	C_5_H_10_O_2_	-	86	-	-	0.26	-
17	Heptanal	C_7_H_14_O	81	90	85	0.19	0.34	2.18
18	Heptenal	C_7_H_12_O	93	95	95	0.53	0.24	0.35
19	5-Methyl furfural	C_6_H_6_O_2_	-	-	87	-	-	0.76
20	(E, E)-2,4-Heptadienal	C_7_H_10_O	86	91	82	0.65	0.50	0.12
21	6-Methylhept-5-en-2-one	C_8_H_14_O	-	92	-	-	0.09	-
22	4-Methylcyclohexanone	C_7_H_12_O	85	-	-	0.18	-	-
23	Heptan-1-ol	C_7_H_16_O	91	92	-	0.07	0.09	-
24	Oct-1-en-3-ol	C_8_H_16_O	-	92	89	-	0.16	0.08
25	1-Hexanoic acid	C_6_H_12_O_2_	85	-	-	0.13	-	-
26	Benzaldehyde	C_7_H_6_O	98	96	98	10.31	3.92	14.2
27	2-ethyl-1-hexanol	C_8_H_18_O	81	-	-	0.13	-	-
28	Octanal	C_8_H_16_O	94	93	95	0.31	0.66	0.50
29	Isononyl alcohol	C_9_H_20_O	84	-	-	0.29	-	-
30	(2E)-2-Octenal	C_8_H_14_O	-	83	88	-	0.29	0.16
31	1-ethenoxy-2,2,4-trimethylpentane	C_10_H_20_O	-	83	-	-	0.11	-
32	Limonene	C_10_H_16_	-	84	-	-	0.10	-
33	Benzyl alcohol	C_7_H_8_O	96	88	97	0.17	0.09	0.32
34	Cineole	C_10_H_18_O	-	80	-	-	0.17	-
35	1-Octanol	C_8_H_18_O	-	91	-	-	0.22	-
36	Citraconic anhydride	C_5_H_4_O_3_	-	-	92	-	-	0.57
37	Phenylacetaldehyde	C_8_H_8_O	90	-	93	0.06	-	1.05
38	4-Isopropylcyclohexanol	C_9_H_18_O	-	82	-	-	0.10	-
39	Nonanal	C_9_H_18_O	94	94	93	1.33	2.01	1.75
40	(2E)-2-Nonenal	C_9_H_16_O	-	93	95	-	0.10	0.09
41	trans, trans-2,4-nonadienal	C_9_H_14_O	-	85	-	-	1.19	-
42	(+)-Isopinocampheol	C_10_H_18_O	-	-	83	-	-	0.11
43	2-Hexylfuran	C_10_H_16_O	-	-	87	-	-	0.08
44	2-decanol	C_10_H_22_O	84	-	-	0.14	-	-
45	Isopulegol	C_10_H_18_O	-	79	-	-	0.97	-
46	Decanal	C_10_H_20_O	92	94	91	0.49	0.28	0.19
47	(2E)-2-Decenal	C_10_H_18_O	-	93	93	-	0.89	0.78
48	(2E,4E)-Deca-2,4-dienal	C_10_H_16_O	90	95	95	0.86	1.20	0.59
49	Undecenal	C_11_H_20_O	91	94	95	1.20	1.08	0.86
50	1-Undecanol	C_11_H_24_O	-	89	83	-	0.07	0.38
51	2-butyl-1-octanol	C_12_H_26_O	84	-	-	0.08	-	-
52	(E,E)-2,4-Dodecadienal	C_12_H_20_O	-	86	-	-	1.76	-
53	Cyclododecane	C_12_H_24_	-	85	-	-	0.17	-
54	Trans-2-dodecen-1-ol	C_12_H_24_O	82	-	-	0.28	-	-
55	4-Undecanolide	C_11_H_20_O_2_	-	81	-	-	0.35	-
56	δ-Undecalactone	C_11_H_20_O_2_	-	83	-	-	0.11	-
57	Trans-2-tridecenal	C_13_H_24_O	89	90	-	1.07	1.14	-
58	1-Pentadecyne	C_15_H_28_	92	80	-	0.26	0.23	-
59	3,7,11-trimethyldodecan-1-ol	C_15_H_32_O	83	85	-	0.28	0.10	-
60	1-Nitrododecane	C_12_H_25_NO_2_	81	-	-	0.06	-	-
61	Tetradecanal	C_14_H_28_O	84	-	-	0.14	-	-
62	Tetradecanal	C_14_H_28_O	-	-	88	-	-	1.17
63	Malonic dihydrazide	C_3_H_8_N_4_O_2_	-	-	80	-	-	0.11
64	z-7-tetradecenal	C_14_H_26_O	89	91	90	0.09	0.59	0.09
65	Diethyl phthalate	C_12_H_14_O_4_	-	97	-	-	0.36	-
66	3-Hydroxydodecanoic acid	C_12_H_24_O_3_	-	82	-	-	0.56	-
67	2-Hexyl-1-decanol	C_16_H_34_O	-	88	-	-	0.06	-
68	(Z)-hexadec-9-enal	C_16_H_30_O	89	94	93	0.41	0.17	0.12
69	(Z)-7-hexadecenal	C_16_H_30_O	80	-	-	2.64	-	-
70	Heptadecan-9-ol	C_17_H_36_O	-	87	-	-	0.21	-
71	Diisobutyl phthalate	C_16_H_22_O_4_	-	83	-	-	0.05	-
72	1-Heptadecanol	C_17_H_36_O	-	-	90	-	-	0.12
73	Palmitic acid	C_16_H_32_O_2_	90	92	94	3.09	4.78	1.98
74	Cyclopentadecanone	C_15_H_28_O	-	88	-	-	0.12	-
75	13-Heptadecyn-1-ol	C_17_H_32_O	86	81	80	0.20	0.16	0.14
76	Octadecanal	C_18_H_36_O	-	93	-	-	0.09	-
77	Dibutyl phthalate	C_16_H_22_O_4_	-	90	-	-	0.05	-
78	Phytol	C_20_H_40_O	-	89	-	-	0.16	-
79	Oleic alcohol	C_18_H_36_O	-	82	-	-	0.23	-
80	(9Z,12Z)-Octadeca-9,12-dien-1-ol	C_18_H_34_O	85	80	83	0.64	0.31	0.31
81	Octadecyl vinyl ether	C_20_H_40_O	-	83	-	-	0.18	-
82	Octadecyl vinyl ether	C_20_H_40_O	-	-	84	-	-	0.46
83	Methyl linoleate	C_19_H_34_O_2_	-	82	83	-	0.81	0.71
84	Methyl linolenate	C_19_H_32_O_2_	83	78	-	0.44	0.41	-
85	Oleyl chloride	C_18_H_33_ClO	91	86	91	0.96	1.92	0.46
86	Linoleoyl chloride	C_18_H_31_ClO	84	88	84	1.74	0.87	0.82
87	Stearic acid	C_18_H_36_O_2_	80	-	89	1.56	-	0.47
88	Oleic acid	C_18_H_34_O_2_	93	94	94	13.49	19.80	12.74
89	Petroselinic acid	C_18_H_34_O_2_	-	-	88	-	-	0.39
90	Linoleic acid	C_18_H_32_O_2_	90	92	95	2.91	4.27	2.60
91	α-Linolenic acid	C_18_H_30_O_2_	-	86	82	-	1.15	0.68
92	Isopropyl linoleate	C_21_H_38_O_2_	-	84	-	-	0.26	-
93	(10Z)-Oxacycloheptadec-10-en-2-one	C_16_H_28_O_2_	80	85	81	1.07	1.60	1.41
94	Hexadecanehydrazide	C_16_H_34_N_2_O	83	-	-	0.16	-	-
95	Prop-2-enyl octadecanoate	C_21_H_40_O_2_	-	85	-	-	0.06	-
96	Octadecanehydrazide	C_18_H_38_N_2_O	84	82	92	0.10	0.20	0.37
97	1-Palmitoyl-rac-glycerol	C_19_H_38_O_4_	-	87	-	-	0.20	-
98	1H-Tetrazole-1-acetic acid	C_3_H_4_N_4_O_2_	84	-	-	0.04	-	-

**Table 2:** Non-volatile components identification of MF samples.

No.	Rt (min)	Precursor Ion (*m*/*z*)	Fragment Ions (*m*/*z*)	Loading Form	Possible Compound	Molecular Formula	Raw MF	MF Pulp	MF Charcoal
1	1.40	138.0546	124.0389 [M+H−CH_2_]^+^, 110.0517 [M+H−CO]^+^	[M+H]^+^	Trigonelline	C_7_H_7_NO_2_	+	−	+
2	1.41	179.0544	152.9980, 132.0313, 111.0097, 96.9699	[M−H]^−^	Glucose	C_6_H_12_O_6_	+	+	+
3	1.47	191.0565	173.0256 [M−H−H_2_O]^−^, 111.0097 [M−H−CO_2_−2H_2_O]^−^	[M−H]^−^	Quinic acid *	C_7_H_12_O_6_	+	+	+
4	1.53	137.0460	119.0453 [M+H−H_2_O]^+^, 110.0244 [M+H−HCN]^+^	[M+H]^+^	Hypoxanthine	C_5_H_4_N_4_O	+	+	−
5	1.54	133.0145	115.0052 [M−H−H_2_O]^−^ 71.0133 [M−H−H_2_O−CO_2_]^−^	[M−H]^−^	Malic acid *	C_4_H_6_O_5_	+	+	+
6	1.54	118.0873	101.0586 [M+H−NH_3_]^+^	[M+H]^+^	Valine	C_5_H_11_NO_2_	+	+	−
7	2.08	136.0665	119.0394 [M+H−NH_3_]^+^, 109.0299 [M+H−HCN]^+^	[M+H]^+^	Adenine	C_5_H_5_N_5_	+	+	−
8	2.34	182.0810	136.0765 [M+H−CH_2_O_2_]^+^, 119.0453 [M+H−CH_2_O_2_−NH_3_]^+^	[M+H]^+^	Tyrosine	C_9_H_11_NO_3_	+	+	+
9	2.36	268.1024	136.0624 [M+H−C_5_H_8_O_4_]^+^	[M+H]^+^	Adenosine	C_10_H_13_N_5_O_4_	−	−	+
10	2.41	132.1019	86.0970 [M+H−CH_2_O_2_]^+^	[M+H]^+^	Leucine	C_6_H_13_NO_2_	+	+	+
11	2.57	115.0037	96.9649 [M−H−H_2_O]^−^, 71.0135 [M−H−CO_2_]^−^	[M−H]^−^	Fumaric acid	C_4_H_4_O_4_	+	+	−
12	2.61	152.0569	135.0298 [M+H−NH_3_]^+^, 110.0289 [M+H−NHCNH]^+^	[M+H]^+^	Guanine	C_5_H_5_N_5_O	+	−	−
13	2.77	191.0157	173.0045 [M−H−H_2_O]^−^, 129.0159 [M−H−H_2_O−CO_2_]^−^, 111.0051 [M−H−2H_2_O−CO_2_]^−^	[M−H]^−^	Citric acid *	C_6_H_8_O_7_	+	+	+
14	3.07	117.0195	99.0081 [M−H−H_2_O]^−^, 73.0288 [M−H−CO_2_]^−^	[M−H]^−^	Succinic acid *	C_4_H_6_O_4_	+	+	+
15	3.35	127.0382	109.0289 [M+H−H_2_O]^+^, 81.0346 [M+H−CH_2_O_2_]^+^, 53.0403 [M+H−C_2_H_2_O_3_]^+^	[M+H]^+^	Pyrogallic acid	C_6_H_6_O_3_	+	+	+
16	3.96	148.0613	102.0531 [M+H−CH_2_O_2_]^+^, 56.0505 [M+H−2C_2_H_2_O_2_]^+^	[M+H]^+^	L−glutamic acid	C_5_H_9_NO_4_	+	−	−
17	4.29	166.0859	120.0807 [M+H−CH_2_O_2_]^+^, 103.0544 [M+H−NH_3_−CH_2_O_2_]^+^	[M+H]^+^	Phenylalanine	C_9_H_11_NO_2_	+	+	+
18	5.63	127.0371	81.0336 [M+H−CO_2_]^+^	[M+H]^+^	5−hydroxymethylfurfural	C_6_H_6_O_3_	+	+	+
19	5.79	153.0148	109.0295 [M−H−CO_2_]^−^	[M−H]^−^	2,5−dihydroxybenzoic acid	C_7_H_6_O_4_	+	+	+
20	5.86	123.0446	96.9652, 87.0090	[M−H]^−^	Guaiacol	C_7_H_8_O_2_	+	−	−
21	5.97	169.0142	125.0267 [M−H−CO_2_]^−^, 107.0179 [M−H−CO_2_−H_2_O]^−^, 97.0292 [M−H−CO_2_−CO]^−^	[M−H]^−^	Gallic acid *	C_7_H_6_O_5_	+	+	−
22	6.03	167.0344	123.0448 [M−H−CO_2_]^−^	[M−H]^−^	Vanillic acid *	C_8_H_8_O_4_	+	+	+
23	6.43	269.0804	254.0635 [M+H−NH_3_]^+^, 237.0519 [M+H−NH_3_−OH]^+^, 118.0498 [M+H−NH_3_−C_7_H_4_O_3_]^+^, 107.0558 [M+H−C_9_H_6_O_3_]^+^	[M+H]^+^	Formononetin	C_16_H_12_O_4_	+	+	−
24	7.34	197.0450	179.0349 [M−H−H_2_O]^−^, 135.0444 [M−H−H_2_O−CO_2_]^−^	[M−H]^−^	Danshensu	C_9_H_10_O_5_	+	+	−
25	7.40	197.0445	169.0110 [M−H−C_2_H_4_]^−^, 124.9769 [M−H−C_2_H_4_−CO_2_]^−^	[M−H]^−^	Ethyl gallate	C_9_H_10_O_5_	+	+	+
26	7.50	156.0772	110.0612 [M+H−CH_2_O_2_]^+^	[M+H]^+^	L−histidine	C_6_H_9_N_3_O_2_	+	−	−
27	7.78	163.0391	119.0507 [M+H−CO_2_]^+^, 107.0495 [M+H−2CO]^+^	[M+H]^+^	7−hydroxycoumarine	C_9_H_6_O_3_	+	+	+
28	8.77	247.0943	147.0433 [M+H−H_2_O−C_4_H_6_−CO]^+^	[M+H]^+^	Columbianetin	C_14_H_14_O_4_	−	+	−
29	11.50	163.0398	119.0494 [M−H−CO_2_]^−^	[M−H]^−^	4−hydroxycinnamic acid	C_9_H_8_O_3_	+	+	−
30	11.94	137.0254	93.0352 [M−H−CO_2_]^−^	[M−H]^−^	Salicylic acid	C_7_H_6_O_3_	+	+	+
31	12.21	353.0874	317.0527 [M−H−2H_2_O]^−^, 191.0563 [M−H−C_9_H_6_O_3_]^−^, 179.0325 [M−H−C_7_H_10_O_5_]^−^, 135.0441 [M−H−C_8_H_10_O_7_]^−^	[M−H]^−^	Neochlorogenic acid *	C_16_H_18_O_9_	+	+	+
32	12.37	271.0674	125.0246 [M−H−C_9_H_6_O_2_]^−^, 119.0494 [M−H−C_7_H_4_O_4_]^−^	[M−H]^−^	Naringenin	C_15_H_12_O_5_	+	+	−
33	12.64	153.0191	109.0293 [M−H−CO_2_]^−^	[M−H]^−^	Protocatechuic acid*	C_7_H_6_O_4_	+	+	+
34	13.44	285.0737	161.0257, 134.0367, 133.0263 [M−H−CO−CO_2_−C_5_H_8_O]^−^	[M−H]^−^	Sappanchalcone	C_16_H_14_O_5_	+	+	+
35	13.47	163.0388	145.0323 [M−H−H_2_O]^−^, 117.0339 [M−H−H_2_O−CO]^−^	[M+H]^+^	7−hydroxycoumarine	C_9_H_6_O_3_	+	+	+
36	13.51	456.1528	323.0997 [M−H−C_8_H_7_NO]^−^	[M−H]^−^	Amygdalin *	C_20_H_27_NO_11_	+	+	+
37	13.95	193.0517	178.0257 [M−H−CH_3_]^−^, 134.0379 [M−H−CH_3_−CO_2_]^−^	[M−H]^−^	Ferulic acid *	C_10_H_10_O_4_	+	+	+
38	13.97	137.0215	108.0222 [M−H−CHO]^−^, 93.0321 [M−H−CO_2_]^−^, 81.0321 [M−H−2CO]^−^	[M−H]^−^	3,4−Dihydroxybenzaldehyde	C_7_H_6_O_3_	+	+	+
39	13.98	179.0352	161.0235 [M−H−H_2_O]^−^, 135.0450 [M−H−CO_2_]^−^	[M−H]^−^	Caffeic acid *	C_9_H_8_O_4_	+	+	+
40	14.09	353.0883	191.0550 [M−H−C_9_H_6_O_3_]^−^, 179.0350 [M−H−C_7_H_10_O_5_]^−^, 135.0445 [M−H−C_8_H_10_O_7_]^−^	[M−H]^−^	Chlorogenic acid *	C_16_H_18_O_9_	+	+	+
41	14.14	353.0876	191.0561 [M−H−C_9_H_6_O_3_]^−^, 173.0448 [M−H−C_9_H_6_O_3_−H_2_O]^−^	[M−H]^−^	Cryptochlorogenic acid *	C_16_H_18_O_9_	+	+	+
42	14.31	121.0296	77.0405 [M−H−CO_2_]^−^	[M−H]^−^	Benzoic acid	C_7_H_6_O_2_	+	+	+
43	14.49	173.0454	155.0346 [M−H−H_2_O]^−^, 137.0254 [M−H−2H_2_O]^−^, 111.0411 [M−H−H_2_O−CO_2_]^−^	[M−H]^−^	Shikimic acid	C_7_H_10_O_5_	+	+	+
44	14.56	109.0257	78.0600, 67.0201	[M−H]^−^	1−(Furan−2−yl) ethenone	C_6_H_6_O_2_	+	+	−
45	14.76	121.0295	108.0236	[M−H]^−^	4−hydroxybenzaldehyde	C_7_H_6_O_2_	+	+	+
46	15.36	165.0546	147.0444 [M+H−H_2_O]^+^	[M+H]^+^	4−hydroxycinnamic acid	C_9_H_8_O_3_	+	+	+
47	15.94	165.0900	137.0589 [M+H−2CH_2_]^+^, 135.0437 [M+H−2CH_3_]^+^, 123.0438 [M+H−C_3_H_6_]^+^	[M+H]^+^	Eugenol	C_10_H_12_O_2_	+	−	−
48	16.17	197.0450	182.0216 [M−H−CH_3_]^−^, 166.9981 [M−H−2CH_3_]^−^	[M−H]^−^	Syringic acid *	C_9_H_10_O_5_	+	+	+
49	16.93	269.0836	161.0273, 145.0255, 117.0306	[M−H]^−^	3−deoxysappanchalcone	C_16_H_14_O_4_	+	−	−
50	18.34	359.1489	200.0962 [M−H−C_7_H_11_O_4_]^−^	[M−H]^−^	(+)−Isolariciresinol	C_20_H_24_O_6_	+	+	−
51	18.78	285.0747	167.0364 [M+H−C_6_H_10_O_5_−C_8_H_5_O]^+^, 151.0407	[M+H]^+^	Glycitein	C_16_H_12_O_5_	+	−	+
52	18.83	279.1595	205.0531 [M+H−C_4_H_10_O]^+^, 149.0232 [M+H−C_4_H_10_O−C_4_H_8_]^+^	[M+H]^+^	Dibutyl phthalate	C_16_H_22_O_4_	−	+	+
53	23.28	515.1180	353.0874 [M−H−C_9_H_6_O_3_]^−^, 335.0770 [M−H−C_9_H_6_O_3_−H_2_O]^−^, 191.0550 [M−H−2C_9_H_6_O_3_]^−^, 179.0358 [M−H−C_9_H_6_O_3_−C_7_H_10_O_5_]^−^, 173.0132 [M−H−2C_9_H_6_O_3_−H_2_O]^−^	[M−H]^−^	Isochlorogenic acid A *	C_25_H_24_O_12_	+	−	+
54	23.34	120.0658	74.0599 [M+H−CO−H_2_O]^+^, 55.9358	[M+H]^+^	Threonine	C_4_H_9_NO_3_	+	−	−
55	23.34	515.1196	353.0858 [M−H−C_9_H_6_O_3_]^−^, 335.0764 [M−H−C_9_H_6_O_3_−H_2_O]^−^, 173.0115 [M−H−2C_9_H_6_O_3_−H_2_O]^−^, 155.0015 [M−H−2C_9_H_6_O_3_−2H_2_O]^−^	[M−H]^−^	Isochlorogenic acid C *	C_25_H_24_O_12_	+	−	+
56	23.41	515.1181	353.0855 [M−H−C_9_H_6_O_3_]^−^, 335.0758 [M−H−C_9_H_6_O_3_−H_2_O]^−^, 191.0549 [M−H−2C_9_H_6_O_3_]^−^, 179.0362 [M−H−C_9_H_6_O_3_−C_7_H_10_O_5_]^−^	[M−H]^−^	Isochlorogenic acid B *	C_25_H_24_O_12_	+	−	+
57	24.89	177.0191	133.0277 [M−H−CO_2_]^−^	[M−H]^−^	Esculetin	C_9_H_6_O_4_	+	+	+
58	24.95	289.0718	271.0603 [M−H−H_2_O]^−^, 245.0796 [M−H−CO_2_]^−^	[M−H]^−^	Catechin	C_15_H_14_O_6_	+	−	−
59	25.52	301.0363	273.0361 [M−H−CO]^−^, 151.0070 [M−H−C_8_H_6_O_3_]^−^	[M−H]^−^	Quercetin*	C_15_H_10_O_7_	+	+	+
60	25.65	463.1014	301.0360 [M−H−C_6_H_10_O_5_]^−^, 300.0290 [M−H−C_6_H_10_O_5_−H]^−^, 271.0274 [M−H−C_6_H_10_O_5_−2H−CO]^−^	[M−H]^−^	Isoquercitrin	C_21_H_20_O_12_	+	+	−
61	25.83	609.1457	463.2785 [M−H−C_6_H_10_O_4_]^−^,301.0349 [M−H−C_6_H_10_O_5_−C_6_H_10_O_4_]^−^	[M−H]^−^	Rutin *	C_27_H_30_O_16_	+	+	+
62	26.19	463.0860	301.0357 [M−H−C_6_H_10_O_5_]^−^	[M−H]^−^	Hyperoside *	C_21_H_20_O_12_	+	+	−
63	26.19	193.0496	178.0255 [M+H−CH_3_]^+^, 165.0543 [M+H−CO]^+^, 150.0313 [M+H−CH_3_−CO]^+^	[M+H]^+^	Scopoletin *	C_10_H_8_O_4_	+	+	+
64	26.32	285.0358	257.0424 [M−H−CO]^−^, 241.0126 [M−H−CO_2_]^−^	[M−H]^−^	Kaempferol	C_15_H_10_O_6_	+	+	+
65	26.57	139.0387	121.0289 [M+H−H_2_O]^+^, 95.0487 [M+H−CO_2_]^+^, 77.0394 [M+H−H_2_O−CO_2_]^+^	[M+H]^+^	4−hydroxybenzoic acid	C_7_H_6_O_3_	+	+	+
66	26.77	285.0404	133.0218 [M−H−C_7_H_4_O_4_]^−^, 107.0179 [M−H−C_8_H_6_O_2_−CO_2_]^−^	[M−H]^−^	Luteolin	C_15_H_10_O_6_	+	+	+
67	27.03	289.0720	245.0813 [M−H−CO_2_]^−^, 203.0705 [M−H−CO_2_−C_2_H_2_O]^−^	[M−H]^−^	Epicatechin	C_15_H_14_O_6_	+	−	−
68	27.69	165.0561	119.0505, 93.0350, 59.0154	[M−H]^−^	Desaminotyrosine	C_9_H_10_O_3_	+	+	−
69	27.80	269.0483	225.0650 [M−H−CO_2_]^−^, 151.0352 [M−H−C_8_H_6_O]^−^, 117.0344 [M−H−C_7_H_4_O_4_]^−^	[M−H]^−^	Apigenin *	C_15_H_10_O_5_	+	+	+
70	27.84	271.0609	253.0442 [M+H−H_2_O]^+^, 243.0643 [M+H−CO]^+^	[M+H]^+^	Genistein	C_15_H_10_O_5_	+	+	+
71	29.70	289.0698	243.0575 [M+H−H_2_O−CO]^+^, 215.0500 [M+H−H_2_O−2CO]^+^, 169.0573 [M+H−2H_2_O−3CO]^+^, 149.0257 [M+H−C_6_H_6_O_2_−CH_2_O]^+^	[M+H]^+^	Eriodictyol	C_15_H_12_O_6_	+	+	+
72	30.79	431.1337	137.0230 [M+H−C_6_H_10_O_5_−C_9_H_8_O]^+^	[M+H]^+^	Ononin	C_22_H_22_O_9_	+	+	+
73	31.22	151.0401	135.0435, 121.0292	[M−H]^−^	Vanillin *	C_8_H_8_O_3_	+	+	+
74	32.67	433.1102	271.0634 [M+H−C_6_H_10_O_5_]^+^	[M+H]^+^	Genistin	C_21_H_20_O_10_	+	+	+
75	33.50	495.1512	195.0677 [M−H−C_6_H_10_O_5_−C_7_H_6_O_3_]^−^, 137.0261 [M−H−C_6_H_10_O_5_−C_10_H_12_O_4_]^−^, 93.0346 [M−H−C_6_H_10_O_5_−C_10_H_12_O_4−_ CO_2_]^−^	[M−H]^−^	Oxypaeoniflorin	C_23_H_28_O_12_	+	−	−
76	33.64	207.0639	192.0464, 177.0165	[M−H]^−^	Ethyl 3−(3,4−dihydroxyphenyl) acrylate	C_11_H_12_O_4_	−	−	+
77	34.28	167.0704	149.0595 [M+H−H_2_O]^+^, 121.0570 [M+H−H_2_O−CO]^+^	[M+H]^+^	Paeonol	C_9_H_10_O_3_	+	+	+
78	34.61	271.0928	167.0381 [M+H−C_8_H_8_]^+^, 121.0631 [M+H−C_8_H_6_O_3_]^+^	[M+H]^+^	Alpinetin	C_16_H_14_O_4_	+	−	−
79	34.75	207.0270	192.0034 [M−H−CH_3_]^−^, 164.0125 [M−H−CH_3−_CO]^−^	[M−H]^−^	Fraxetin	C_10_H_8_O_5_	+	+	−
80	36.28	274.2735	106.0848, 102.0909, 88.0760, 70.0662	[M+H]^+^	Lauryl diethanolamine	C_16_H_35_NO_2_	−	−	+
81	38.98	469.3321	423.3246 [M−H−CH_2_O_2_]^−^	[M−H]^−^	Camaldulenic acid	C_30_H_46_O_4_	+	+	+
82	39.04	227.2010	149.0463, 59.0153	[M−H]^−^	Myristic acid	C_14_H_28_O_2_	+	−	−
83	39.09	503.3233	184.0722, 57.0708	[M+H]^+^	Medicagenic acid	C_30_H_46_O_6_	+	−	−
84	40.05	279.2335	261.2230 [M−H−H_2_O]^−^	[M−H]^−^	Linoleic acid	C_18_H_32_O_2_	+	+	−
85	40.34	487.3424	85.0274 [M−H−C_26_H_42_O_3_]^−^	[M−H]^−^	Tormentic acid	C_30_H_48_O_5_	+	+	+
86	41.73	471.3486	224.0697, 455.3221	[M−H]^−^	Maslinic acid *	C_30_H_48_O_4_	+	+	+
87	42.60	471.3468	224.0734, 455.3342	[M−H]^−^	Corosolic acid *	C_30_H_48_O_4_	+	+	+
88	45.98	455.3542	407.3389 [M−H−CH_2_O_2_−2H]^−^, 391.2542 [M−H−CH_2_O_2_−H_2_O]^−^	[M−H]^−^	Oleanic acid	C_30_H_48_O_3_	+	+	+
89	46.56	455.3539	409.3590 [M−H−CH_2_O_2_]^−^	[M−H]^−^	Ursolic acid	C_30_H_48_O_3_	+	+	+

*: The compound was identified by the standard.

**Table 3:** The classification results by discriminant analysis.

Batch	Actual Groups	Predictive Groups	Discriminant Scores
R1	1	1	−3.03239
R2	1	1	−2.56128
R3	1	1	−1.82510
R4	1	1	−3.58109
R5	1	1	−2.17700
R6	1	1	−4.07484
R7	1	1	−0.63608
R8	1	1	−4.39140
R9	1	1	−2.46782
R10	1	1	−2.56013
R11	1	1	−2.01371
R12	1	1	−2.41586
P1	2	2	0.41325
P2	2	2	0.21712
P3	2	2	1.96516
P4	2	2	1.99673
P5	2	2	−0.13852
P6	2	2	1.44538
P7	2	2	−0.38917
P8	2	2	0.19001
P9	2	2	0.62724
P10	2	2	−0.11257
P11	2	2	0.01797
P12	2	2	0.60738
C1	3	3	2.69825
C2	3	3	2.30163
C3	3	3	1.90670
C4	3	3	0.96356
C5	3	3	3.02390
C6	3	3	2.49040
C7	3	3	1.43859
C8	3	3	0.56368
C9	3	3	−0.04875
C10	3	3	2.99477
C11	3	3	3.77244
C12	3	3	2.79154
R13	-	1	−3.23409
R14	-	1	−2.26340
R15	-	1	−2.85846
R16	-	1	−2.70495
R17	-	1	−3.09759
P13	-	2	−1.12422
P14	-	2	−0.20967
P15	-	2	−2.24227
P16	-	2	−3.58321
P17	-	2	−0.79827
C13	-	3	0.82540
C14	-	3	1.44190
C15	-	1 *	0.03417
C16	-	3	2.31326
C17	-	3	2.31503

1, represented the raw MF group; 2, represented the MF pulp group; 3, represented the MF charcoal groups; -, represented the unknown groups; * represented the incorrect classification.

**Table 4:** Mass spectra properties of 16 analytes using UHPLC-MS/MS analysis.

Compounds	Prec. Ion (*m*/*z*)	Prod. Ion (*m*/*z*)	Frag. (V)	C.E. (V)	Ion Mode
Succinic acid	117.0	73.1	75	12	Negative
L-malic acid	133.0	115.1	70	8	Negative
3,4-Dihydroxybenzaldehyde	137.0	108.0	118	28	Negative
Protocatechuic acid	153.0	109.0	98	16	Negative
Caffeic acid	179.0	135.0	88	16	Negative
D-quinic acid	191.0	93.0	136	24	Negative
Citric acid	191.0	111.0	80	8	Negative
Ferulic acid	193.1	134.0	93	16	Negative
Syringic acid	197.0	182.0	98	12	Negative
Cryptochlorogenic acid	353.1	173.1	113	16	Negative
Neochlorogenic acid	353.1	191.0	113	20	Negative
Chlorogenic acid	353.1	191.0	103	12	Negative
Amygdalin	456.1	323.1	176	8	Negative
Maslinic acid	471.4	471.4	275	39	Negative
Corosolic acid	471.4	471.4	305	5	Negative
Rutin	609.1	300.0	219	40	Negative

Note: Prec Ion: precursor ion; Prod Ion: product ion; Frag: fragmentor; CE: collision energy.

## Data Availability

All datasets presented in this study are included in the article/Supplementary Materials.

## Supplementary Materials

The following supporting information can be downloaded at: https://www.mdpi.com/article/10.3390/molecules27196344/s1, Figure S1: TIC diagrams of raw MF, MF pulp, and MF charcoal in GC-MS analysis; Figure S2: The OPLS-DA loading plots of three groups MF samples using GC-MS analysis by 487 variables, the most important compounds with VIP > 1 are highlighted in red bulleted dot (**A**), 19 differential volatile chemical markers are highlighted in red bulleted dot (**B**); Figure S3: TICs of raw and processed MF in positive (**A**) and negative (**B**) ions using UHPLC-Q-TOF-MS/MS analysis; Figure S4: The OPLS-DA loading plots of three groups MF samples using UHPLC-Q-TOF-MS/MS analysis by 2986 variables, the most important compounds with VIP > 1 are highlighted in red bul-leted dot (**A**), 16 potential differential markers are highlighted in red bulleted dot (**B**); Figure S5: MRM chromatograms of succinic acid (1), L-malic acid (2), 3,4-Dihydroxybenzaldehyde (3), protocatechuic acid (4), caffeic acid (5), D-quinic acid (6), citric acid (7), ferulic acid (8), syringic acid (9), cryptochlorogenic acid (10), neochlorogenic acid (11), chlorogenic acid (12), amygdalin (13), maslinic acid (14), corosolic acid (15), rutin (16). (A) standard solution; (B) MF sample; Table S1: The RSDs of precision, repeatability, and stability in GC-MS analysis; Table S2:The accuracy of different variables by metabolomics methods; Table S3: The RSDs of precision, repeatability, and stability in UHPLC-Q-TOF-MS/MS analysis; Table S4: Linear equation, linear range, correlation coefficients (r), lower LOQ, and lower LOD of 16 investigated analytes in UHPLC-MS/MS analysis (*n* = 6); Table S5: RSDs of precision, repeatability, stability, and recovery of 16 compounds in UHPLC-MS/MS analysis (*n* = 6); Table S6: REs and RSDs of dilution effect of 16 compounds in UHPLC-MS/MS analysis (*n* = 6); Table S7: The contents of 16 compounds in MF samples (μg/g, *n* = 3); Table S8. The origin of seventeen raw MF samples.

## Sample Availability

The *Mume Fructus* samples are available from the authors.
