# Supplementary material for: Comparative Investigation of the Differences in Chemical Compounds between Raw and Processed Mume Fructus Using Plant Metabolomics Combined with Chemometrics Methods
Source: Molecules. 2022 Sep 26;27(19):6344. doi: 10.3390/molecules27196344 (PMC9572716; doi:10.3390/molecules27196344)
Supplement: Supplementary file 1 [file molecules-27-06344-s001.zip › molecules-1865078-supplementary.pdf]

molecules

Supplementary Materials

Comparative Investigation of the Differences in Chemical Compounds between Raw and Processed *Mume Fructus* Using Plant Metabolomics Combined with Chemometrics Methods

Songrui Wang, Shujie Wei, Yameng Zhu, Mengmeng Zhang, Xiunan Cao, Yanxu Chang, Huizi Ouyang * and Jun He *

State Key Laboratory of Component-based Chinese Medicine, Tianjin University of Traditional Chinese Medicine, Tianjin 301617, China

* Correspondence: huihui851025@163.com (H.O.); hejun673@tjutcm.edu.cn (J.H.); Tel./Fax: +86-22-59596163 (J.H.)

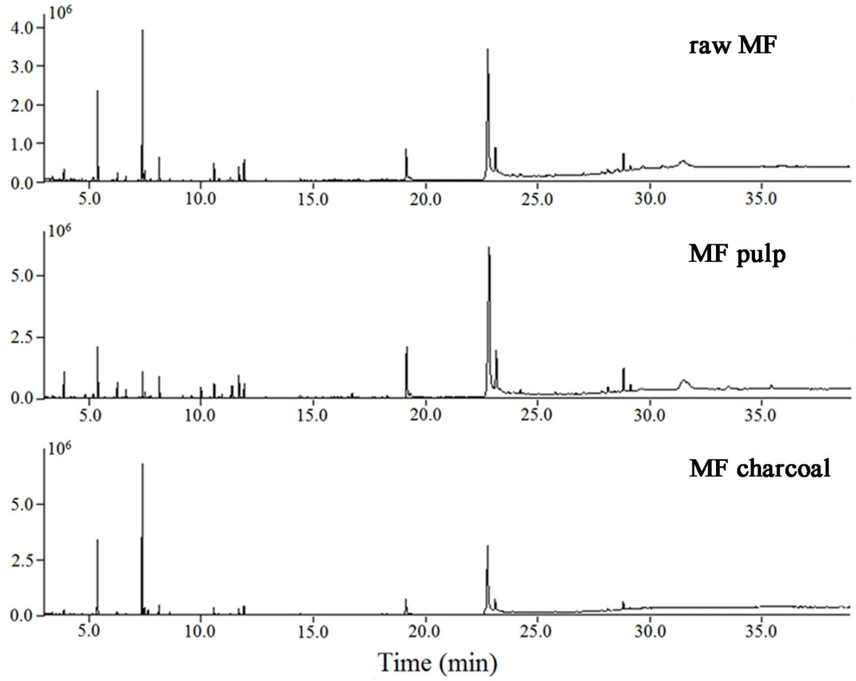

Figure S1. TIC diagrams of raw MF, MF pulp, and MF charcoal in GC-MS analysis.

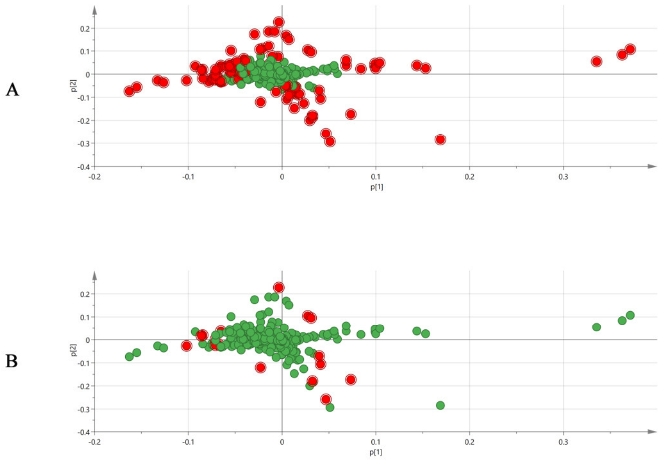

Figure S2. The OPLS-DA loading plots of three groups MF samples using GC-MS analysis by 487 variables, the most important compounds with VIP > 1 are highlighted in red bulleted dot (**A**), 19 differential volatile chemical markers are highlighted in red bulleted dot (**B**).

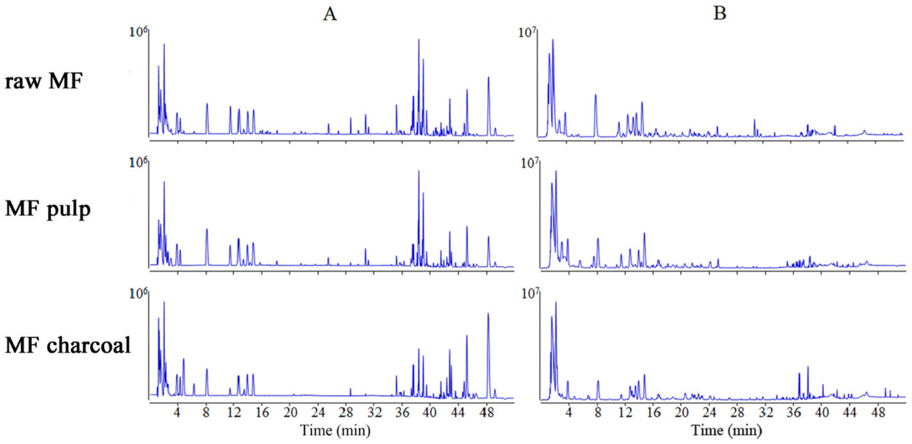

Figure S3. TICs of raw and processed MF in positive (**A**) and negative (**B**) ions using UHPLC-Q-TOF-MS/MS analysis.

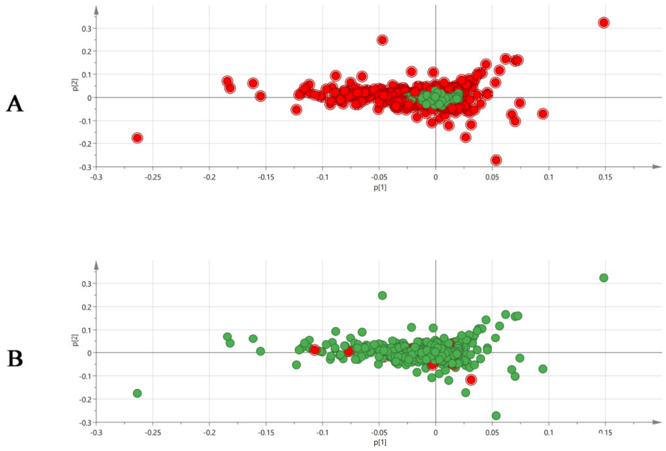

Figure S4. The OPLS-DA loading plots of three groups MF samples using UHPLC-Q-TOF-MS/MS analysis by 2986 variables, the most important compounds with VIP > 1 are highlighted in red bulleted dot (**A**), 16 potential differential markers are highlighted in red bulleted dot (**B**).

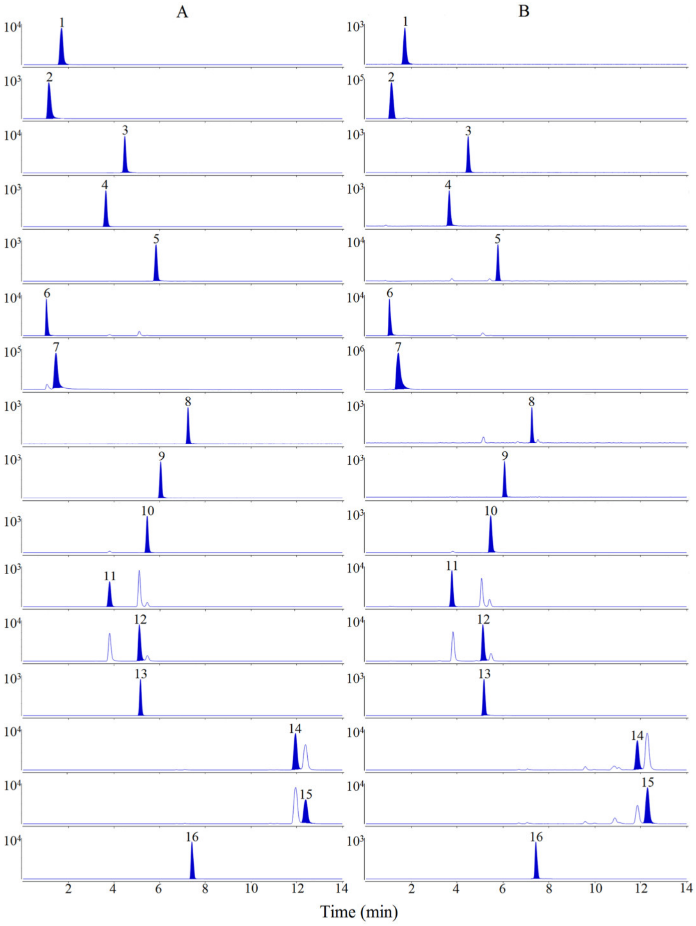

Figure S5. MRM chromatograms of succinic acid (1), L-malic acid (2), 3,4-Dihydroxybenzaldehyde (3), protocatechuic acid (4), caffeic acid (5), D-quinic acid (6), citric acid (7), ferulic acid (8), syringic acid (9), cryptochlorogenic acid (10), neochlorogenic acid (11), chlorogenic acid (12), amygdalin (13), maslinic acid (14), corosolic acid (15), rutin (16). (**A**) standard solution; (**B**) MF sample.

Table S1. The RSDs of precision, repeatability, and stability in GC-MS analysis.

Peak	Precision RSD (%)		Repeatability RSD (%)		Stability RSD (%)	
	Rt	Peak Area	Rt	Peak Area	Rt	Peak Area
1	0.10	5.53	0.06	4.84	0.15	7.54
2	0.05	5.85	0.02	6.13	0.05	6.84
3	0.03	6.03	0.01	6.81	0.05	7.65
4	0.23	5.30	0.06	6.41	0.26	7.29
5	0.31	5.48	0.06	6.22	0.36	4.95
6	0.03	7.04	0.07	9.12	0.04	7.24
7	0.40	7.60	0.04	6.87	0.65	4.64
8	0.04	5.87	0.01	5.13	0.04	5.50
9	0.03	8.08	0.02	5.81	0.03	6.12
10	0.02	4.52	0.01	8.20	0.03	3.75
11	0.01	6.14	0.01	6.86	0.03	7.08
12	0.02	5.98	0.03	6.47	0.02	8.13
13	0.01	7.11	0.01	8.08	0.01	8.02
14	0.06	5.48	0.43	8.12	0.04	7.74
15	0.06	5.16	0.08	4.62	0.07	4.84
16	0.02	6.88	0.29	8.39	0.01	6.02
17	0.01	8.71	0.01	8.64	0.02	6.85
18	0.19	8.72	0.12	7.85	0.08	2.90
19	0.04	6.52	0.03	8.44	0.04	6.94
20	0.02	8.04	0.01	4.80	0.02	5.27

Table S2. The accuracy of different variables by metabolomics methods.

Metabolomics Methods	Variables	Accuracy
GC-MS analysis	487	100%
	99	88.75%
	19	85.72%
Positive ion modes in UHPLC-Q-TOF-MS/MS analysis	3605	100%
	674	81.08%
	12	79.33%
Negative ion modes in UHPLC-Q-TOF-MS/MS analysis	2986	100%
	420	94.37%
	26	80.87%
	16	96.68%

Table S3. The RSDs of precision, repeatability, and stability in UHPLC-Q-TOF-MS/MS analysis

Peak	Precision RSD (%)		Repeatability RSD (%)		Stability RSD (%)	
	Rt	Peak Area	Rt	Peak Area	Rt	Peak Area
1	0.85	4.03	0.97	2.52	0.90	3.48
2	0.70	5.66	0.58	5.53	0.69	6.95
3	0.51	4.04	0.53	6.44	0.48	4.13
4	0.49	2.63	0.47	3.16	0.46	2.88
5	0.44	3.34	0.43	2.04	0.40	4.09
6	0.40	3.14	0.45	2.45	0.37	3.14
7	0.40	4.99	0.39	2.77	0.37	5.21
8	0.32	3.08	0.38	2.61	0.29	3.26
9	0.27	3.36	0.34	2.40	0.24	4.04
10	0.23	3.13	0.30	3.22	0.21	3.44
11	0.22	3.17	0.28	3.96	0.15	2.79
12	0.19	1.85	0.25	6.56	0.13	4.52
13	0.17	2.40	0.22	2.86	0.11	6.50
14	0.16	2.55	0.23	5.45	0.10	3.59
15	0.15	4.82	0.19	2.96	0.07	6.09
16	0.13	5.21	0.15	2.53	0.11	5.77
17	0.11	5.96	0.18	6.88	0.01	5.59
18	0.14	4.52	0.13	6.49	0.03	6.59
19	0.03	6.75	0.05	1.84	0.01	5.97
20	0.03	4.99	0.05	5.28	0.02	6.99

Table S4. Linear equation, linear range, correlation coefficients (r), lower LOQ, and lower LOD of 16 investigated analytes in UHPLC-MS/MS analysis (*n* = 6).

Compound	Linear Equation	Linearity Range (ng/mL)	r	LLOQ (ng/mL)	LLOD (ng/mL)
Succinic acid	Y=166.6736X − 324.8084	5–2500	0.9992	2.68	0.80
L-Malic acid	Y=209.3703X − 346.7145	50–25000	0.9992	2.42	0.73
3,4-	Y=167.6880X − 270.9234	2–1000	0.9992	0.48	0.14
Protocatechuic acid	Y=631.6267X − 1367.9688	5–2500	0.9996	2.63	0.79
Caffeic acid	Y=1150.3923X+750.3464	2–1000	0.9995	0.73	0.22
D-Quinic acid	Y=25.8069X+227.6506	50–25000	0.9994	9.39	2.82
Citric acid	Y=197.1403X − 4723.7186	100–50000	0.9993	40.19	12.06
Ferulic acid	Y=113.0834X − 101.2560	2–1000	0.9994	0.13	0.04
Syringic acid	Y=97.2186X − 54.5327	2–1000	0.9991	0.26	0.08
Cryptochlorogenic acid	Y=305.6614X+32.2740	20–10000	0.9992	4.81	1.44
Neochlorogenic acid	Y=937.0648X+2720.3225	25–12500	0.9992	2.17	0.65
Chlorogenic acid	Y=1481.6196X+2744.2515	20–10000	0.9997	1.65	0.50
Amygdalin	Y=84.8160X − 697.7015	40–20000	0.9993	2.89	0.87
Maslinic acid	Y=2378.6398X − 3077.2010	2–1000	0.9996	0.22	0.06
Corosolic acid	Y=1776.5292X+714.7926	2–1000	0.9995	0.20	0.06
Rutin	Y=557.5183X − 171.7546	2–1000	0.9995	0.27	0.08

Table S5. RSDs of precision, repeatability, stability, and recovery of 16 compounds in UHPLC-MS/MS analysis (n = 6).

**Compound**	**Precision (RSD, %)**		**Repeatability (RSD, %)**	**Stability (RSD, %)**	**Sample Recovery**	
	**Intra-day**	**Inter-day**			**Average Recovery Rate (%)**	**RSD (%)**
Succinic acid	1.70	2.81	2.36	1.82	98.46	3.37
L-Malic acid	2.57	3.75	2.77	4.17	89.90	5.92
3,4-Dihydroxybenzaldehyde	6.77	5.84	5.19	5.32	94.38	5.98
Protocatechuic acid	0.83	3.85	3.84	2.05	98.20	3.39
Caffeic acid	1.80	3.22	2.48	3.09	105.90	3.05
D-Quinic acid	1.10	1.77	0.70	1.08	101.14	1.31
Citric acid	3.41	1.96	2.57	1.73	97.73	1.77
Ferulic acid	4.00	6.07	5.18	2.77	105.02	4.89
Syringic acid	3.98	5.21	5.90	6.90	108.26	3.96
Cryptochlorogenic acid	0.79	1.06	1.50	0.99	101.38	2.60
Neochlorogenic acid	2.55	1.46	1.33	0.96	103.95	3.61
Chlorogenic acid	2.02	2.78	1.48	2.42	99.70	2.46
Amygdalin	0.63	1.18	3.24	1.57	110.45	6.12
Maslinic acid	4.72	3.93	2.41	1.31	94.29	6.35
Corosolic acid	5.46	5.41	3.06	3.29	94.13	5.03
Rutin	2.02	4.97	2.94	3.97	88.81	2.63

Table S6. REs and RSDs of dilution effect of 16 compounds in UHPLC-MS/MS analysis (n = 6).

Compounds	Dilution Times	Theoretical Value (ng/mL)	Measured Value (ng/mL)	RE (%)	RSD (%)
succinic acid	20	1250	1272.04 ± 16.74	1.76	1.32
	50	500	471.62 ± 21.40	−5.68	4.54
	100	250	237.22 ± 13.22	−5.11	5.57
L-malic acid	20	12500	12462.08 ± 323.18	−0.30	2.59
	50	5000	5222.13 ± 202.56	4.44	3.88
	100	2500	2493.43 ± 63.79	−0.26	2.56
3,4-Dihydroxybenzaldehyde	20	500	505.18 ± 12.90	1.04	2.55
	50	200	210.24 ± 5.03	5.12	2.39
	100	100	99.46 ± 5.56	−0.54	5.59
protocatechuic acid	20	1250	1199.37 ± 40.99	−4.05	3.42
	50	500	470.21 ± 14.13	−5.96	3.01
	100	250	261.50 ± 8.13	4.60	3.11
caffeic acid	20	500	491.12 ± 22.63	−1.78	4.61
	50	200	191.77 ± 7.20	−4.12	3.75
	100	100	99.71 ± 4.32	−0.29	4.33
D-quinic acid	20	12500	12939.36 ± 313.38	3.51	2.42
	50	5000	5253.08 ± 170.37	5.06	3.24
	100	2500	2576.38 ± 98.03	3.06	3.81
citric acid	20	25000	24146.58 ± 1274.09	−3.41	5.28
	50	10000	9626.59 ± 294.39	−3.73	3.06
	100	5000	4978.67 ± 337.79	−0.43	6.78
ferulic acid	20	500	479.90 ± 8.91	−4.02	1.86
	50	200	187.97 ± 6.35	−6.01	3.38
	100	100	96.02 ± 4.23	−3.98	4.41
syringic acid	20	500	496.52 ± 11.29	−0.70	2.27
	50	200	185.42 ± 5.85	−7.29	3.15
	100	100	97.11 ± 5.66	−2.89	5.83
cryptochlorogenic acid	20	5000	4795.71 ± 28.72	−4.09	0.60
	50	2000	1878.02 ± 54.26	−6.10	2.89
	100	1000	982.02 ± 63.66	−1.80	6.48
neochlorogenic acid	20	6250	6185.59 ± 57.48	−1.03	0.93
	50	2500	2427.11 ± 62.66	−2.92	2.58
	100	1250	1257.16 ± 21.17	0.57	1.68
chlorogenic acid	20	5000	5034.20 ± 144.42	0.68	2.87
	50	2000	2113.77 ± 74.72	5.69	3.53
	100	1000	969.21 ± 44.97	−3.08	4.64
amygdalin	20	10000	9919.19 ± 500.45	−0.81	5.05
	50	4000	4180.00 ± 126.78	4.50	3.03
	100	2000	2090.93 ± 79.08	4.55	3.78

	20	500	467.18 ± 7.07	−6.56	1.51
maslinic acid	50	200	185.38 ± 5.92	−7.31	3.19
	100	100	104.60 ± 5.63	4.60	5.39
	20	500	507.34 ± 8.61	1.47	1.70
corosolic acid	50	200	209.92 ± 5.66	4.96	2.70
	100	100	100.24 ± 6.95	0.24	6.94
	20	500	495.48 ± 14.10	−0.90	2.85
rutin	50	200	193.87 ± 12.73	−3.06	6.57
	100	100	103.00 ± 3.65	3.00	3.54

Table S7. The contents of 16 compounds in MF samples (μg/g, *n* = 3).

Compounds	Sort	Batch																
		1	2	3	4	5	6	7	8	9	10	11	12	13	14	15	16	17
Succinic acid	Raw	92.7	90.3	75.0	82.2	103.0	58.5	131.7	59.3	116.8	84.6	71.6	115.6	69.6	116.1	101.8	67.6	64.3
	Pulp	129.5	97.4	119.8	109.3	132.4	100.6	174.8	81.7	109.1	105.0	99.5	142.6	101.2	102.7	109.9	90.3	112.3
	Charcoal	118.9	107.7	91.4	116.5	118.6	94.0	222.5	38.6	108.6	101.6	103.6	118.3	89.7	105.0	99.2	74.1	121.7
L-Malic acid	Raw	4032.8	5755.0	2354.1	4650.1	3962.0	2649.5	8528.3	1139.6	4682.2	4782.3	4986.9	2458.5	2425.8	6831.4	4359.8	4626.4	4393.6
	Pulp	7043.3	6116.3	4424.6	6678.7	8372.0	5487.9	11421.9	4938.6	5215.8	7928.2	7102.4	3719.7	4884.9	5848.7	5767.7	5113.9	5002.5
	Charcoal	5577.1	5011.9	3490.2	6126.4	6013.7	6799.3	7211.1	1435.9	5581.5	6412.0	6917.3	3255.4	3798.5	4762.1	5752.0	3800.5	4345.9
3,4-Dihydroxybenzaldehyde	Raw	5.3	23.0	14.8	17.2	6.8	6.3	32.2	18.3	19.2	12.1	11.9	12.0	12.8	55.3	22.5	15.6	18.2
	Pulp	25.5	23.7	43.7	48.3	25.6	42.1	58.3	35.5	13.1	32.4	24.4	47.8	33.4	32.6	44.4	49.7	45.2
	Charcoal	10.1	13.9	15.4	15.2	12.2	14.5	17.8	26.9	12.2	13.3	15.6	22.7	16.2	29.7	14.6	17.2	31.3
Protocatechuic acid	Raw	21.2	36.5	35.6	38.7	20.7	23.0	26.9	64.2	39.5	34.2	32.9	138.1	25.2	106.1	45.3	42.0	79.1
	Pulp	42.1	37.9	47.7	56.8	41.3	53.4	25.2	60.3	31.1	42.9	35.7	224.9	36.3	86.9	58.1	66.3	107.3
	Charcoal	27.1	39.3	43.5	43.8	27.6	42.9	26.3	81.1	27.7	34.0	38.5	201.3	34.9	103.1	45.7	56.6	92.6
Caffeic acid	Raw	78.8	82.1	74.1	72.4	79.5	54.8	58.2	85.6	53.2	113.1	65.1	57.3	42.1	85.1	78.5	81.6	69.8
	Pulp	142.8	88.4	92.4	106.0	147.2	95.2	70.0	106.0	51.3	154.6	71.4	70.8	61.4	75.0	89.6	114.6	73.4
	Charcoal	71.6	53.0	49.0	47.1	72.7	72.9	36.5	90.9	45.9	82.0	55.3	55.0	41.7	60.3	39.9	84.8	76.6
D-Quinic acid	Raw	4875.2	4084.4	4828.6	3570.0	5002.2	4112.4	1434.0	6337.2	3805.7	5481.9	4342.5	4890.8	3159.5	4116.8	5323.6	3970.7	4594.1
	Pulp	6290.3	5540.2	5839.1	5081.5	5934.3	6080.5	1521.5	6264.8	5635.7	5820.2	4749.4	7211.5	4715.8	6341.1	6402.3	6653.7	6371.8
	Charcoal	4439.8	4056.2	4630.0	3441.7	4264.9	4137.9	1405.0	5177.5	2967.4	4190.0	3347.3	4812.5	2844.8	3903.8	4874.3	5169.4	4434.8
Citric acid	Raw	204633.2	268941.9	243851.5	290604.9	193752.0	186558.9	277264.8	206457.0	212895.5	256561.1	250425.8	191280.6	189849.1	225758.1	229726.2	250185.5	227378.2
	Pulp	399719.8	386443.0	371023.2	359769.6	429190.4	378431.7	345451.6	350159.7	394997.8	421893.9	386281.4	214203.8	344755.7	394031.8	322619.5	361327.8	391807.7
	Charcoal	201542.2	221687.5	172169.6	203956.5	198846.7	231223.9	184411.2	208761.6	206201.7	219424.8	181823.3	133180.4	180549.0	174561.3	162215.5	204943.2	158265.0

Ferulic acid	Raw	17.7	20.8	19.5	17.0	18.5	13.0	25.5	22.6	13.8	20.5	16.7	14.2	11.5	19.8	13.7	10.9	13.2
	Pulp	24.4	19.1	26.5	27.1	23.3	22.9	28.7	22.4	14.5	26.2	25.0	19.9	17.2	16.7	16.7	24.8	18.5
	Charcoal	22.8	15.8	20.1	18.3	31.5	20.6	23.9	25.2	18.3	23.0	21.7	14.8	14.7	20.6	9.4	21.2	20.6
Syringic acid	Raw	26.2	36.3	42.1	32.7	25.2	22.5	44.4	29.8	38.6	29.4	36.7	25.2	41.8	42.4	34.9	32.4	28.8
	Pulp	48.9	53.8	69.2	60.3	45.2	65.4	47.9	50.3	41.8	44.3	51.8	44.2	56.2	40.0	50.8	59.3	46.3
	Charcoal	37.3	46.3	57.3	41.7	42.3	39.1	52.3	43.1	38.6	39.5	46.7	39.2	51.8	42.4	41.8	46.0	42.3
Cryptochlorogenic acid	Raw	2473.9	2591.9	2549.2	2288.5	2885.3	2216.4	1223.4	2694.0	2242.5	2711.0	2160.5	1620.2	2014.1	2178.6	2647.1	2434.3	1959.3
	Pulp	3568.8	2942.7	3207.9	3286.5	3438.5	3323.3	1341.0	3455.2	2807.8	3580.8	2493.3	2254.6	2845.4	2731.6	3246.3	3248.8	2653.3
	Charcoal	2510.4	2206.1	2055.2	1966.9	2680.4	2494.0	978.1	2736.8	2158.0	2539.1	2171.0	1659.4	1767.0	1972.5	1790.3	2661.3	2630.2
Neochlorogenic acid	Raw	3992.7	2928.6	3107.2	2367.0	4468.0	2864.3	1667.3	2960.8	2731.0	3632.2	2507.1	1175.7	2027.0	2183.4	3122.4	2672.2	2165.7
	Pulp	5734.5	4277.1	4925.1	3925.7	5807.3	4594.4	2159.3	4576.0	3798.8	5418.0	3546.2	1863.5	3721.9	3438.4	4087.8	4598.6	3234.0
	Charcoal	3279.1	2296.8	2167.8	1792.7	3458.0	2746.3	1136.5	3001.2	2583.6	2888.8	2090.6	1221.4	1611.4	1958.1	1762.0	3020.4	2717.9
Chlorogenic acid	Raw	2665.8	1814.0	1810.3	1620.5	2743.0	1729.9	1832.0	1593.1	1722.8	2486.0	1684.4	1075.3	1923.0	1436.2	1806.8	1703.2	1492.5
	Pulp	3283.5	2389.4	2838.9	2767.7	3330.7	2601.1	2414.2	3201.9	2303.3	3378.5	2458.8	1651.7	2830.6	2185.7	2357.8	2431.3	2184.1
	Charcoal	2646.2	2013.3	1723.3	1623.6	2394.2	2212.9	1387.1	2088.7	1815.4	2533.1	1944.4	1301.3	1919.6	1795.5	1531.7	2081.2	2014.1
Amygdalin	Raw	4779.6	1414.5	2781.8	2053.2	3804.6	4423.1	8856.7	4385.9	4499.6	798.0	2575.2	1781.9	3602.1	2480.3	3649.8	3103.3	271.3
	Pulp	149.5	186.7	78.6	52.7	135.3	80.7	260.3	75.7	40.8	88.9	166.8	270.7	29.5	59.2	93.4	119.7	101.6
	Charcoal	356.3	676.8	1440.9	603.0	202.2	541.7	9210.6	2876.7	5680.6	928.5	1089.6	2539.2	1016.3	542.8	657.6	1535.3	522.9
Maslinic acid	Raw	79.1	57.0	73.3	53.9	64.6	49.3	150.8	68.8	94.1	57.0	47.2	88.4	71.0	96.1	64.2	55.2	44.6
	Pulp	111.5	82.9	140.0	96.9	105.6	107.7	180.8	121.0	104.8	123.1	101.0	182.0	137.5	118.2	135.1	114.1	135.0
	Charcoal	52.7	54.7	84.1	54.8	68.7	65.8	160.0	56.1	72.9	55.0	41.2	89.9	55.6	85.1	62.6	49.5	70.9
Corosolic acid	Raw	194.7	184.0	164.1	184.0	201.0	136.6	226.4	153.8	189.3	216.7	158.6	183.2	204.9	204.1	210.5	182.6	130.9
	Pulp	317.6	263.8	288.7	302.3	308.0	308.6	257.5	347.8	202.5	360.9	313.6	396.6	359.6	267.4	343.7	371.9	335.2
	Charcoal	189.1	174.8	167.4	153.0	200.3	193.5	187.2	173.7	153.8	178.0	139.3	212.6	164.6	181.5	142.0	168.1	206.2
Rutin	Raw	154.7	74.2	63.8	49.6	152.5	50.8	12.9	85.7	75.6	105.0	51.0	19.0	77.5	25.0	60.5	38.4	41.1
	Pulp	169.5	83.9	105.2	100.9	178.1	87.2	12.0	155.5	97.7	144.4	72.6	40.4	121.6	66.2	92.9	103.0	44.2
	Charcoal	76.3	32.5	32.6	28.8	71.2	53.1	6.8	54.5	73.7	84.3	39.0	16.4	35.2	30.8	22.9	44.5	31.9

Table S8. The origin of seventeen raw MF samples.

Batch	Origin		Lot Number
1	Yunnan	Dali City	20200911
2		Dali City	20200926
3		Kunming City	20201013
4		Kunming City	20200930
5		Dali City	20201011
6	Sichuan	Dazhou City	20201006
7		Chengdu City	20200906
8		Chengdu City	20201012
9		Chengdu City	20201008
10		Dazhou City	20201017
11		Leshan City	20200928
12	Xinjiang	Wulumuqi City	20200731
13		Wulumuqi City	20200823
14		Wulumuqi City	20200910
15		Wulumuqi City	20200923
16	Anhui	Bozhou City	20200925
17		Bozhou City	20200820
